# Supplementary material for: Exploring how lifestyle weight management programmes for children are commissioned and evaluated in England: a mixed methodology study
Source: BMJ Open. 2019 Dec 16;9(12):e025423. doi: 10.1136/bmjopen-2018-025423 (PMC6937081; doi:10.1136/bmjopen-2018-025423)
Supplement: Supplementary data [file bmjopen-2018-025423supp001.pdf]

University of BRISTOL

Supplementary File 1 – Online Survey Questions:

National Survey of LA Commissioned Weight Management Services for Overweight / Obese Children in 2014/15

	**Informed Consent for Online Survey**
	In order to take part in this online survey, we need to obtain your informed consent. Please read the following five statements carefully.<br>1. I confirm that I have read and understood the participant information.<br>2. I am willing to take part in the survey<br>3. I understand that my name will be kept anonymous however due to the nature of the study, it is not possible to anonymise the local authority name<br>4. I understand that information collected (name of survey participant anonymised but name of local authority not anonymised) will be stored for 10 years in data sets within a secure facility in accordance with the Data Protection Act 1998 and this data may be used in publications or presentations to relevant audiences or shared with other researchers.<br>5. I understand that I can withdraw from the study at any point prior to March 21st 2016 by emailing Dr Ruth Mears on rm14101@bristol.ac.uk
**Question 1**	Please confirm that you have read, understood and agree to the above five statements
**Response 1**	
	**Participant & Local Authority Details**
**Question 2**	What local authority do you work in?<br>*Please note that if you work for multiple local authorities, you will need to fill out a new survey for each local authority that you work for.*
**Response 2**	
	**Tier 2 Weight Management Services for Overweight/Obese Children in your Local Authority**
**Question 3**	Please name a Tier 2 weight management service commissioned by your local authority for overweight / obese children aged 4-16 years during the financial year April 2014-March 2015?
**Response 3**	
**The following questions relate to the Tier 2 weight management service you have named above.**	
**Question 4**	What evidence are you aware of regarding the effectiveness of the service commissioned between April 2014 – March 2015 at reducing BMI centile / BMI % / BMI z-score or BMI? Please choose (highlight) from the below list;
**Response 4**	– *Data published in a peer reviewed journal – independently collected (i.e. data collected by a person who is NOT an employee of the weight management service provider)*

	– *Data published in a peer reviewed journal – internally collected (i.e. data collected by a person who IS an employee of the weight management service provider)*<br>– *Published in an alternative source – independently collected (i.e. data collected by a person who is NOT an employee of the weight management service provider)*<br>– *Published in an alternative source – internally collected (i.e. data collected by a person who IS an employee of the weight management service provider)*<br>– *Unpublished data*<br>– *Other*<br>– *No evidence*
**Question 5**	Please specify where the evidence can be found regarding the effectiveness of the services commissioned during the year April 2014-March 2015 at reducing BMI centile / BMI % / BMI z-score or BMI?<br>*(e.g. publication details / website address etc. If the data is unpublished, please email details to rm14101@bristol.ac.uk)*
**Response 5**	
**Question 6**	Since the service was commissioned (i.e. contract start date), had it been evaluated within your local authority?
**Response 6**	
**Question 7**	As part of the service evaluation, was change in weight status measured (e.g. change in BMI, BMI%, BMI centile or BMI z-score?)
**Response 7**	
**Question 8**	If change in weight status was measured, what were the results?<br>*Please write the time frame in which this change occurred e.g. Reduction of BMI centile by 0.9% (SD) over 1 year (2014/15). If this is available for different age groups, please indicate the results by age group.*
**Response 8**	
**Question 9**	What was the total cost of the service for the local authority between April 2014 to March 2015?<br>*If data cannot be provided please specify the time period and costs in the format you have available e.g. cost per child per course in August 2014.*
**Response 9**	
**Question 10**	What was the maximum number of participants that could have been accommodated by the commissioned service between April 2014 to March 2015?<br>*Where possible, please provide data on maximum commissioned capacity for a one year time frame from 2014 to 2015. If this data cannot be provided, please specify the time period and maximum capacity of the service in the format you have available e.g. maximum capacity of 60 children per course in 2014, total of 10 courses in 2014.*

**Response 10**	
**Question 11**	How many children were referred to the service between April 2014 to March 2015?<br>*If this data cannot be provided please specify the time period and referral data in the format you have available*
**Response 11**	
**Question 12**	How many children completed the intervention between April 2014 to March 2015?<br>*If data cannot be provided please specify the time period and number completing the intervention in the format you have available*
**Response 12**	
	**Thank you for taking the time to complete this survey. Please consider taking part in the second phase of our research.**
**Question 13**	Thank you for taking the time to complete this survey.<br><br>Would you like to receive a summary of the results and analysis by email?<br><br>If you answered yes to the above question, please provide us with your email address
**Response 13**	
**Question 14**	If you answered yes to the above question, please provide us with your email address
**Response 14**	
	**Telephone Interview**

The second phase of our research will involve a telephone interview exploring commissioners' views and experiences in the evaluation of weight management services for overweight and obese children. There is little qualitative evidence available regarding service evaluation data collected by commissioners and this research aims to fill the gap in the literature. We will explore the views of commissioners on the role and value of service evaluation data, the barriers and facilitators to collecting and processing this data and finally how to ensure evaluation data is useful. Performance management of services will also be explored.

If you are interested in participating in the interview, please can you provide your name and contact details (email and/or telephone number) below. We will then email you a participant information sheet providing further details about what the interview involves. After reading the information sheet, if you decide you would like to take part, you will need to fill out the consent form and send it to rm14101@bristol.ac.uk. Dr Ruth Mears will then contact you to arrange a convenient time for you to conduct the telephone interview

………
………
………

University of BRISTOL
