# Supplementary material for: Exploring how lifestyle weight management programmes for children are commissioned and evaluated in England: a mixed methodology study
Source: BMJ Open. 2019 Dec 16;9(12):e025423. doi: 10.1136/bmjopen-2018-025423 (PMC6937081; doi:10.1136/bmjopen-2018-025423)
Supplement: Supplementary data [file bmjopen-2018-025423supp002.pdf]

Supplementary File 2

Interview Guide: Commissioning and evaluation of lifestyle weight management programmes in England

**Icebreaker**	**Prompts**
What is your role in the local authority?	*How long have you been in this role?*
How are you involved in the commissioning of weight management services for obese and overweight children?	*What is your background? Public Health?*
**Part One: Role and value of evaluations**	
What do you understand by the term 'service evaluation'?	*Why conduct a service evaluation? Role for commissioners? Role for participants? Role for service providers?*
What do you understand by the term 'performance management'?	*Why collect performance management data?*
How do you think service evaluation and performance management differ?	
How important do you think service evaluation is?	*Importance to commissioners / LA / personal opinions? Why? What informs the decision to undertake a service evaluation (e.g. pilot, new service, lack of evidence, review, retenderin)?*
How useful do you think service evaluation is?	*Why? What parts are useful? What parts are not useful? Do your personal views differ from what you feel are the views of the local authority?*
What outcomes are currently viewed as a measure of success by your local authority for a childhood weight management programme?	*Do you agree that this outcome should be viewed as a measure of success? What are your personal views? Which outcomes do you think are the most important? Why?*
What outcome data from a service evaluation is viewed as essential by your local authority? *(or performance management data if service evaluations have not been conducted)*	*Why? Do you agree?*
What information from a service evaluation does your local authority least value? *(or performance management data if service evaluations have not been conducted)*	*Why? Do you agree?*

Supplementary File 2

**Part Two: LA Specific Questions**	
Reflecting on a weight management service you have been involved in, can you tell me how the service was evaluated?<br>*If the service has not been evaluated, please can you tell me about performance management of the service*.	*Who collected the evaluation data (external evaluator or internal person)? At what time points was the data collected? What data was collected? Was a specific evaluation data collection tool used?*
What went well in collecting evaluation data *(or performance management data if no service evaluation has been conducted)*?	*What barriers were there? What are the weaknesses in your data? What are the strengths of your data? Were service providers happy to co-operate with the evaluation process? Were there any difficulties in collecting data from service users? How could service evaluation data be improved?*
What did not go well in collecting evaluation data *(or performance management data if no service evaluation has been conducted)*?	
Do you think the data collected was useful?	***Yes:*** *In what way was it useful? How could it have been more useful? How were the data used?*<br>***No:*** *Why do you think it was not useful? Is there are anything which would have made it more useful?*
How were the service evaluation data *(or performance management data if no service evaluation has been conducted)* used by your local authority?	*By commissioners? By service providers? To improve services? To inform future commissioning decisions? Is this reflective of how other service evaluation data has been used*
How do you think the information collected from a service evaluation *(or performance management data if no service evaluation has been conducted)* should be used for maximum benefit?	*By commissioners? By service providers?*
**Part Three: Improving the process of service evaluation and use of outcome data**	
What resources / tools / information / guidance are currently available to help commissioners conduct service evaluations?	*Are they useful? What are the downsides of them? What would you find useful to have which is not currently available?*
Do you have access to online journals - OVID / Medline databases etc?	*Would you feel comfortable reviewing evidence from these databases?*
Who is responsible for collecting the service evaluation *(or performance management)* outcome data in your local authority?	*If it is the service providers, do you think they should be? Why? Why not?*
What are your opinions on the sharing of evaluation *(or performance data)* between local authorities and other organisations?	*Do you have any reservations? Do you think it would be beneficial or*

Supplementary File 2

	detrimental? Use in future commissioning decisions?
Do you share evaluation *(or performance data)* with other local authorities or organisations?	***Yes:*** *How do you do this? Is the data actively shared? Is it useful? Is the data shared of good quality? Does it play a role in future commissioning decisions?*<br>***No:*** *Why not? Do you think data should be shared? What are the barriers to sharing data?*
Do you think service contracts should be based on performance?	*E.g. must attain x change in BMI percentile or the contract will be terminated. What are the benefits of linking service contracts to performance? What are the downsides of this?*
In your Local Authority, are service evaluations used to identify underperforming services?	***Yes:*** *Do you think they should be used in this way? If a weight management service within your local authority is underperforming, are there any procedures which you would follow?*<br>***No:*** *Do you think they should be used in this way?*
How do you think the process of service evaluation *(or performance data)* and use of outcome data could be optimised to ensure maximum benefit for commissioners?	*Maintaining standards? Future decisions? Sharing?*
Are you aware of any recent national (NOO or NICE) guidelines regarding the evaluation of lifestyle weight management programmes?	*What do you know about them? At what time points do these guidelines recommend collecting outcome data? Does your local authority currently collect data at this timepoint? Is your local authority planning to implement these guidelines?*
What information do you think is needed to assist commissioners in providing successful weight management programmes?	*(Prompts; research? evidence? guidelines?)*
**Closing**	
That's all the questions I have for you today. Do you have any other comments you wish to make about service evaluations?	
Do you have any questions for me?	
Thank you very much for your time and attention. We appreciate you sharing your thoughts and opinions with us.	
