# Supplementary material for: Exploring how lifestyle weight management programmes for children are commissioned and evaluated in England: a mixed methodology study
Source: BMJ Open. 2019 Dec 16;9(12):e025423. doi: 10.1136/bmjopen-2018-025423 (PMC6937081; doi:10.1136/bmjopen-2018-025423)
Supplement: Author's manuscript [file bmjopen-2018-025423.draft_revisions.pdf]

BMJ Open is committed to open peer review. As part of this commitment we make the peer review history of every article we publish publicly available.

When an article is published we post the peer reviewers' comments and the authors' responses online. We also post the versions of the paper that were used during peer review. These are the versions that the peer review comments apply to.

The versions of the paper that follow are the versions that were submitted during the peer review process. They are not the versions of record or the final published versions. They should not be cited or distributed as the published version of this manuscript.

BMJ Open is an open access journal and the full, final, typeset and author-corrected version of record of the manuscript is available on our site with no access controls, subscription charges or pay-per-view fees (http://bmjopen.bmj.com).

If you have any questions on BMJ Open's open peer review process please email editorial.bmjopen@bmj.com

BMJ Open

Lifestyle weight management programmes in childhood: how are they commissioned and evaluated?

Journal:	*BMJ Open*
Manuscript ID	bmjopen-2018-025423
Article Type:	Research
Date Submitted by the Author:	19-Jul-2018

Complete List of Authors:	Mears, Ruth; Centre for Academic Primary Care, Bristol Medical School, University of Bristol,; Centre for Exercise Nutrition and Health Sciences, School for Policy Studies, University of Bristol,<br>Jago, Russ; Centre for Exercise Nutrition and Health Sciences, School for Policy Studies, University of Bristol,; National Institute for Health Research (NIHR) Collaboration for Leadership in Applied Health Research and Care West (CLAHRC West) at University Hospitals Bristol NHS Foundation Trust,<br>Sharp, Deborah; Centre for Academic Primary Care, Bristol Medical School, University of Bristol,<br>Sharma, Anamica; Centre for Exercise Nutrition and Health Sciences, School for Policy Studies, University of Bristol,<br>Kipping, Ruth; Department of Population Health Sciences, Bristol Medical School, University of Bristol,; Centre for the Development and Evaluation of Complex Interventions for Public Health Improvement (DECIPHer),<br>Shield, Julian; NIHR Bristol Biomedical Research Centre, Nutrition Theme, University of Bristol,; School of Clinical Sciences, University of Bristol,

Keywords:	obesity, lifestyle weight management services, children, commissioning, evaluation

SCHOLARONE™
Manuscripts

Lifestyle weight management programmes in childhood: how are they commissioned and evaluated?

Mears R [1,2], Jago R [2,3], Sharp D [1], Sharma A[2], Kipping R [4,5], Shield JPH [6,7]

Centre for Academic Primary Care, Bristol Medical School, University of Bristol, BS8 2PS, UK [1]. Centre for Exercise Nutrition and Health Sciences, School for Policy Studies, University of Bristol, BS8 1TZ, UK [2]. National Institute for Health Research (NIHR) Collaboration for Leadership in Applied Health Research and Care West (CLAHRC West) at University Hospitals Bristol NHS Foundation Trust, BS1 3NU[3]. Department of Population Health Sciences, Bristol Medical School, University of Bristol, BS8 2PS, UK [4]. Centre for the Development and Evaluation of Complex Interventions for Public Health Improvement (DECIPHer), Bristol, BS8 2PS, UK [5]. NIHR Bristol Biomedical Research Centre, Nutrition Theme, University of Bristol, BS1 2NT, UK [6]. School of Clinical Sciences, University of Bristol, BS2 8DZ, UK [7].

Corresponding Author: rm14101@bristol.ac.uk

Author Positions: Dr Ruth Mears – NIHR Academic Clinical Fellow in Primary Care. Professor Russell Jago - Professor of Paediatric Physical Activity and Public Health. Professor Debbie Sharp - Professor of Primary Health Care. Ms. Anamica Sharma - MSc Student. Ms. Ruth Kipping - Senior Research Fellow in Epidemiology and Public Health. Professor Hamilton-Shield - Professor in Diabetes and Metabolic Endocrinology.

For peer review only

Abstract

Objectives: To assess how lifestyle weight management programmes for children aged 4-16 years in England are commissioned and evaluated at the local level.

Design: This was a mixed methodology study comprising of an online survey and semi-structured telephone interviews.

Setting: An online survey was sent to all Local Authorities (LAs) in England regarding lifestyle weight management services commissioned for children aged 4-16 years. Online survey data were collected between February and May 2016 and based on services commissioned between April 2014 and March 2015. Semi-structured telephone interviews with LA staff across England were conducted between April and June 2016.

Participants: Commissioners or service providers working within the Public Health Department of LAs.

Main outcome measures: The online survey collected information on the evidence-base, costs, reach, service usage and evaluation of child lifestyle weight management services. The telephone interviews explored the nature of child weight management contracts commissioned by LAs, the type of outcome data collected and whether these data were shared with other LAs or organisations, the challenges faced by these services and the perceived 'markers of success' for a programme.

Results: The online survey showed that none of the participating LAs were aware of any peer-reviewed evidence supporting the effectiveness of their specific commissioned service. Despite this, the telephone interviews revealed that there was no national formal sharing of data to enable oversight of the efficacy of commissioned services across LAs in England to help inform future commissioning decisions. Challenges with long-term data collection, service engagement, funding and the pressure to reduce the prevalence of obesity were frequently mentioned.

Conclusions: Consideration needs to be given as to whether evidence-based, population-level interventions should be given preferential funding rather than small-scale lifestyle weight management services with unclear robust evidence of clinically significant changes in weight status and uncertain long-term effectiveness.

Strengths and limitations of this study

- There have been no previous independent peer-reviewed research studies assessing how lifestyle weight management programmes in childhood are being commissioned and evaluated across LA's in England.
- The response rate for the online survey was lower than desired however there was good geographical representation across England and some lack of response may be attributed to LA's not commissioning lifestyle weight management services for children.
- The current study only focused on LAs in England so generalisation of results to the rest of the UK and wider is unclear.

For peer review only - http://bmjopen.bmj.com/site/about/guidelines.xhtml

Introduction

In the past four decades, there has been a tenfold increase in the number of obese children and adolescents worldwide [1 2]. In the United Kingdom, 31.1% of children and adolescents were classified as overweight or obese in 2016 [3]. These children and adolescents are more likely to become overweight or obese adults and suffer health related consequences [4]. This presents a major public health challenge [5]. In the UK, weight management strategies are classified into tier one (those that focus on preventing obesity), tier 2 (lifestyle weight management services), tier 3 (specialist obesity services) and tier 4 services (pharmacological or surgical treatments for obesity). [6] Tier 1 and 2 services are commissioned by public health departments working within Local Authorities (LAs). Tier 3 and 4 services are commissioned by a combination of clinical commissioning groups (CCG's) and NHS England[7].

This paper focuses on tier 2 weight management services commissioned by Local Authorities across England for school-aged children (aged 4-16 years). There are 152 Local Authorities in England [8] and each LA may choose to commission a different tier 2 service provider. Although there is guidance from the National Institute of Clinical Excellence (NICE) and Public Health England (PHE) regarding what these services should comprise of [6 9], the specific weight management programmes developed by the different service providers have rarely been independently evaluated and published. Furthermore, there are very few UK-based randomised trials in the peer-reviewed literature demonstrating a clinically significant reduction in BMI z-score (defined as minimum BMI SDS reduction of ≥ 0.25)[10 11] through lifestyle weight management programmes alone for school aged-children [12 13 14]. Even the evidence reviews supporting the NICE guidance PH47, only reported a post-intervention pooled reduction in BMI z-score of -0.17 (95% CI = -0.3 to -0.04, p=0.01) which was attenuated when long-term data (≥ 6 months) were used (SMD = -0.07; CI 95% = −0.15 to 0.02, p = 0.12) [15].

Local Authorities usually monitor their tier 2 weight management services through 'Performance Management' meetings, although they may also conduct service evaluations. NICE recommends that monitoring focuses on sustaining long term changes [6], despite their evidence reviews showing little efficacy for these interventions in the long-term [15]. Given the poor evidence-base for tier 2 weight management services, it is important to understand more about the nature of the contracts commissioned by Local Authorities, the monitoring of outcomes and the challenges facing these services. In addition, given the current financial climate in public health, with the ring-fenced public health budget in Local Authorities lifted in 2019, it is important to explore whether these services are a good use of limited resources.

This mixed methodology study uses quantitative methods (an online survey) to determine the evidence-base, costs, reach, service usage and evaluation of tier 2 weight management programmes commissioned by Local Authorities across England for children aged 4-16 years. Qualitative methods (semi-structured telephone interviews) explore the nature of childhood tier 2 weight management contracts commissioned by LAs, the type of outcome data collected and whether these data are shared, the challenges faced by these services

For peer review only - http://bmjopen.bmj.com/site/about/guidelines.xhtml

and the perceived ‘markers of success’ for a programme. Finally, the data collected from both the online survey and telephone interviews examine whether lifestyle weight management programmes are a good use of limited resources.

For peer review only

For peer review only - http://bmjopen.bmj.com/site/about/guidelines.xhtml

Methods

Participants and Recruitment

A list of all LAs in England was derived from 2014/2015 National Child Measurement Programme (NCMP) datasets [8]. The Director for Public Health for each LA was contacted by email and asked to identify the relevant person within their LA responsible for the commissioning of childhood tier 2 weight management services. An email was sent to this person asking if they would be willing to participate in an online survey exploring tier 2 weight management services for school-aged children commissioned between April 2014 and March 2015. If no response to the email was received, a further email was sent and follow up telephone calls made.

The final page of the online survey provided information about the second phase of the study (telephone interviews) and invited those interested in taking part to leave their contact details. In addition, some of those Local Authorities who declined to participate in the online survey, were also invited by email to take part in the telephone interviews.

Ethical Approval

Ethical approval was granted by the School for Policy Studies Research Ethics Committee at the University of Bristol. Informed consent was obtained in written format for the online survey and in written or verbal format for the telephone interviews.

Online Survey

The online survey consisted of 10 questions relating to tier 2 weight management services commissioned by the Local Authority for overweight or obese children aged 4-16 years in March 2014 to April 2015 (See Supplementary File 1). The survey collected data on the evidence-base supporting the commissioned intervention, the cost of the service, the maximum number of participants the service could have accommodated, the number of children referred, the number of children completing the intervention, whether a service evaluation had been conducted and the changes in weight status measured through service evaluation. Data were collected between February and May 2016 and analysed in Microsoft Excel.

Telephone Interviews

Semi-structured telephone interviews were conducted by RM between April and June 2016. The interview guide was developed by RM, RJ, DS, JHS and RK. The interview guide had a common framework but was adapted during the interview as guided by the participants' responses.

The interviews required participants to reflect on their experiences of tier 2 weight management services for school-aged children within their Local Authority but was not confined to experiences within the time-period specified in the online survey of March 2014-April 2015. This enabled a broader representation of experiences from interview participants. The interviews explored the nature of the contracts commissioned by Local

For peer review only - http://bmjopen.bmj.com/site/about/guidelines.xhtml

Authorities and the monitoring of these services through performance management and service evaluation. Specifically, the interviews explored whether outcome data were collected, whether these data were shared, the challenges identified through monitoring processes and the perceived 'markers of success' for the service.

Interviews were audio-recorded then transcribed verbatim by Bristol Transcription Service. All interview transcripts were anonymised by AS and uploaded to N-vivo 10.0 for inductive thematic analysis.

Data were organised into codes and themes and constantly revised and reviewed by two researchers working independently (RM and AS). Once coding was complete, both researchers discussed differences and cohesions within and across themes before agreeing on the final themes. The themes reflected the questions asked during the interviews and topics most explored by the participants.

The final sample size was determined by the saturation of information when no new information seemed to emerge. This resulted in a final sample of 20 participants.

Transparency statement

The online survey was conducted as originally planned. The telephone interviews initially aimed to explore service evaluation and performance management of tier 2 weight management services for children from a commissioner's perspective and experiences. As it emerged that some Local Authorities run in-house contracts, participants were also included who were within a Local Authority but service providers. The data which subsequently emerged focused the analysis on determining whether lifestyle weight management programmes were a good use of limited resources.

For peer review only - http://bmjopen.bmj.com/site/about/guidelines.xhtml

Results

Quantitative Data from Online Survey

Survey Respondents

Sixty-four Local Authorities responded to our request to participate in the online survey. Of these, forty completed the online survey and twenty-four declined to participate. The reasons given by the latter group were; nil commissioned (n=14), service decommissioned (n=4), insufficient resources to complete the online survey (n=3) and declined for other reasons (n=3).

Geographical Location of Survey Respondents

The geographical location of the forty Local Authorities who completed the online survey were; North West (n=10), North East (n=2), Yorkshire and the Humber (n=4), West Midlands (n=3), East Midlands (n=1), East of England (n=3), London (n=7), South West (n=7) and South East (n=3). The population of children aged 4-16 years within each of these forty Local Authorities ranged from 16,000 to 186,000 (Mid-2014 Population Data from Office of National Statistics).

Evidence-base of Tier 2 Weight Management Service Commissioned

No Local Authorities were aware of evidence published in peer-reviewed journals demonstrating that their service was effective at improving BMI centile (or other weight related measure). Service evaluations were conducted in 55% of Local Authorities, of which 18% did not measure change in weight status as part of their service evaluation. Due to heterogeneity in the way in which outcome data for change in weight status were reported by Local Authorities, it was not possible to make any meaningful interpretations or comparisons of these data.

Costs and Reach of the Service

Table 1 summarises the costs of the service. Some Local Authorities were only able to provide estimates. Table 2 summarises the reach of services within an LA.

Table 1: Costs of the Service

	Mean cost (SD, n = number of Local Authorities providing data)
Cost of the service per year to Local Authority	£130,742 (SD £122,869, n=27)
Cost of the service per year per 10,000 children aged 4-16 years (of any weight) in Local Authority	£29,396 (SD £30,003, n=27)
Cost of the service per overweight or obese child attending if maximum capacity of the service was reached	£558 (SD £408, n=18)
Cost of the service per child completing the intervention	£1,312 (SD £1342, n= 15)

For peer review only - http://bmjopen.bmj.com/site/about/guidelines.xhtml

Table 2: Reach of the Service

	Mean (SD, n)
Potential reach of the service (presuming maximum capacity was achieved) to overweight or obese children within a LA	3.5% (SD 6.9%, n=26) *
Estimated actual reach of the service (i.e. children completing the intervention) to overweight or obese children within a LA	1.2% (SD 1.6%, n=25) *

** These calculations used estimates of the prevalence of overweight or obese children within a LA aged 4-16 years (this was estimated using NCMP data from Reception and Year 6 and National Statistics population data for children aged 4-16 years).*

Qualitative Data from Telephone Interviews

Twenty telephone interviews were conducted with Local Authorities (18 commissioners, 2 service providers within the Local Authority). Seventeen of the telephone interview participants had completed the online survey. Three had declined. The geographical location of the twenty Local Authorities who completed the interview were; North West (n=8), North East (n=1), Yorkshire and the Humber (n=1), West Midlands (n=0), East Midlands (n=1), East of England (n=1), London (n=4), South West (n=3) and South East (n=1). Interviews were between 28 and 68 minutes in length.

Nature of Commissioning Contracts

Tier 2 weight management contracts were either between the Local Authority and an external provider, or 'in-house' contracts (where the local authority acts as both the commissioner and service provider). Some Local Authorities reported running 'in-house' contracts as they could not afford to commission the service to an external provider. This was not a problem if the service was performing well, however if the service was underperforming, their options might be limited as they may not be able to go out to market due to financial and political pressures.

- **INT 3**: *'if they're not achieving their targets, they're not doing their job properly, so then we shouldn't be providing the service, but what is the alternative? It's too expensive to commission it out'*

One local authority discussed the challenges of 'in-house' contracts from a leadership perspective, especially as their service was not meeting BMI targets.

- **INT 17:** *'To make it complicated our provider is also within the local authority so there's a bit of – it's something that provides such a huge challenge just on its own because you've got provider senior leadership and commissioning senior leadership with different views'……… 'the service underachieved against the targets around BMI consistently over the last two years……..If they were an external provider it would probably be a different scenario'*

For peer review only - http://bmjopen.bmj.com/site/about/guidelines.xhtml

Outcome Data

All Local Authorities collected outcome data through performance management processes and some also collected outcome data through service evaluation. Most interventions were around 12 weeks long with data collected at baseline and at the end of the intervention. Some Local Authorities also attempted to collect longer-term data at 3 month, 6 months and/or 12 months. Although the general themes of data collected were similar (demographic data, retention, engagement, weight, self-esteem, confidence, behavioural change, physical activity, diet), the actual data were collected in different formats across some Local Authorities. For example, some Local Authorities measured physical activity via a seven-day recall questionnaire, others through a physical activity test and others by asking parents whether their children increased their activity levels or through assessing physical literacy. There were some outcome measures unique to one or two Local Authorities e.g. **INT 5:** '*improved confidence with portion size'* or **INT 11:** *'the percentage of parents who have increased their confidence to read food labels from baseline'.*

Challenges identified through Service Evaluation and/or Performance Management Meetings

1. **Lack of long-term data**

Many participants mentioned the difficulties in collecting long-term follow-up data. This was attributed to a variety of factors including length of questionnaires, lack of parental confidence with the paperwork, too much effort for families to undertake, people moving around town, resource constraints of LA to capturing this data, lack of IT infrastructure and lack of engagement in both the intervention and the evaluation.

- **INT 14:** *'It becomes then quite time consuming to try and chase patients who engaged. People forget what they've done 12 months ago or more as well. …………it would be quite difficult with not having things like a GP surgeries infrastructure like EMIS where data gets held for years and years and it's there to use and accessed again'.*

2. **Lack of validated tools**

Some participants felt that there was a lack of validated tools to enable accurate outcome measures to be obtained.

- **INT 1:** *'We're looking for validated tools but there are just not that many great ones out there.'*

3. **Reliability of self-report data**

A few participants questioned the reliability of self-report data.

For peer review only - http://bmjopen.bmj.com/site/about/guidelines.xhtml

- **INT 3:** *'Other challenges are self-reporting. …. The physical activity and nutrition tend to be improved after ten weeks and sometimes you look on that a little cynically because the measurements haven't improved, so perhaps they're telling us what we want to hear, that can be a challenge'.*

4. **Lack of engagement**

Difficulties engaging children, parents and healthcare professionals with the service was mentioned by many of the Local Authorities. This is summarised in Table 3.

Table 3: Challenges of Engaging Parents, Children and Health-Care Professionals with the service

Difficulties engaging parents	
Talking about the weight of a child can be highly emotive for parents;	**INT 19:** *'It's difficult with parents sometimes to explain to them that what they are doing at home is probably not the best thing for their child. That's quite difficult you know, that's their baby that's their child and they don't want to hear anything negative.'*
Parents often find it difficult to accept that their child is overweight;	**INT 5:** *'Parents often see their children as normal weight when they are in fact overweight and we know people often will refer to children who are a normal weight as a bit skinny,'*
Parents often do not recognise the role they need to play in engaging in the service as part of a 'family intervention' to improve their child's BMI centile	**INT 11:** *'So we say it has to be a family intervention. But they don't always see it that way. They just want the child to lose the weight and don't acknowledge their role in being the providers' food and the environment they grow up in'.*
Difficulties engaging children	
Engaging children with the service could be challenging;	**INT 2:** *'there is a lot of issues around recruitment and retentions with tier two services for children and also there's a great difficulty with actually the secondary aged children to get them sort of accessing services'.*
Difficulties engaging healthcare professionals	
Healthcare professionals can find it difficult to bring up weight status of a child with a parent	**INT 4:** *'I think there's definitely issues there from what I've heard about professionals bringing things up with families'*
Some healthcare professionals fail to	**INT 5:** *'The GP will look at the child and say, it's just puppy fat, they'll grow out of it'.*

For peer review only - http://bmjopen.bmj.com/site/about/guidelines.xhtml

recognise overweight or obese children	**INT 11:** *'We even get some out of school nurses say 'well, they're only just into the overweight category'. You know, the child is really athletic, they're really muscular'*
Lack of GP engagement	**INT 10:** *'GP's still struggle to engage with it'*<br>**INT 6:** *'GPs, locally they tend not to refer'*

5. Lack of resources / expertise

A few commissioners felt that service providers lacked expertise in conducting service evaluations.

- **INT 14:** *'there's difficulties there with the data that we need because we also find that the skill set of a lot of the people delivering the services doesn't always sit with evaluation'.*

Financial Pressures on Services

There are considerable financial pressures facing Local Authorities at present and budget constraints are impacting on the provision of tier 2 weight management services for children in most Local Authorities in different ways.

- **INT 17:** *'we're at a point now where we're going through council budget savings, the service has actually taken a 50% hit, which is huge'.................' so how are we supposed to achieve this whole you know like city wide target on less money is going to be impossible'.........'We've got smaller and smaller services and you keep telling me you're going to take some more money away from me so how are we supposed to achieve these things'*

Some Local Authorities have found it challenging to provide a good service with reduced funding. Strategies taken to cope with the funding cuts have included setting lower targets as part of the key performance indicators (KPIs).

- **INT 1:** *'we've had to work together to reduce the KPIs anyway because they just wouldn't be met with that much of a dent in the finances'*

A few Local Authorities are considering, or have already decided, to decommission their weight management service.

- **INT 2:** *'. So yeah things are really tight and at the regional network meeting people were talking that they may have to de-commission their weight management services'.*

Local Authorities talked about the need to demonstrate 'good value for money' for a service to justify funding of the service.

For peer review only - http://bmjopen.bmj.com/site/about/guidelines.xhtml

- **INT 5:** *'I'm constantly looking at a cost benefit analysis and working out, okay how much is this costing per child, how much is it costing per family? What are the outcomes that we're getting? Is this really a programme that is cost effective?'*

A few Local Authorities discussed the difficulties in allocating money to service evaluation when money for service provision itself was so limited.

Pressures on Service to Influence the Prevalence of Obesity

Local Authorities often described the pressures they are under to reduce the prevalence of obesity within their borough through their tier 2 weight management programme. In some Local Authorities, this seemed to be politically driven by councillors.

- **INT 2: '***They're fixated about our actual prevalence rate'…..'the councillors yeah and sort of senior management. We've got like sort of corporate score card and they wanted to put obesity prevalence as part of that.'*

Reducing the prevalence of obesity was frequently seen as an unrealistic goal given the reach of the service often being so small, the funding allocated limited and the feeling that one service cannot be accountable for solely tackling such a complex problem with a programme length that is usually only 10-12 weeks.

- **INT 14;** *'In terms of tackling childhood obesity I'd say the child weight management programmes are family weight management programmes, they're only going to go so far. We know our population in LA14, we've probably got 500 families within each year group that would be affected by obesity even more that would be affected by overweight. If you times that by 18 years of childhood you've got quite a significant number of families up in the 10,000 maybe that are going to have these weight management issues. We're never going to be able to commission a service that would be able to work at a one to one level or a group level with 10,000 families, it's not going to be practical to do that. On the other side of things, we're looking at strategies that take a much more preventative approach.'*

To achieve the objective of reducing the prevalence of obesity, some Local Authorities recognised that population-based approaches would be required.

- **INT 15: '***the number of people we're getting to is actually quite small …….. it's not going to change obesity levels locally, so we do need to look at more population-based approaches so that's something we will be doing... I suppose doing less programmes possibly in future because the numbers per programme aren't as high as we'd want'*

For peer review only - http://bmjopen.bmj.com/site/about/guidelines.xhtml

Need for a 'Whole Systems Approach'

Many of the Local Authorities talked about a recent shift towards a 'whole systems approach' to tackling obesity and the need to view weight management schemes alongside the 'bigger picture'.

- **INT 2:** *'we can run weight management schemes and I think they're really important, but it has to be part of the bigger picture because you know children's families only go to those sort of schemes like once a week. It's their whole environment that it's important to actually help them to making behaviour change and actually if we don't do both and try and change the obesogenic environment people aren't going to be successful in weight management and it's only going to be a short term, isn't it'*

Some felt that national strategies to try and change the obesogenic environment (e.g. active transport, sugar tax, change for life campaign) and perception of what constitutes a healthy weight were needed to influence the prevalence of obesity.

- **INT 3:** *'It's not going to be easy because it's more and more difficult to make healthy living the norm because it's just too easy to be unhealthy. It's going to take a major upheaval for it to get any better. I think the sugar taxes could help, I think we're going to see more and more of these. What I think we could do to improve it is more and more national campaigns, that's what I think'.*

Sharing and Use of Evaluation Data

Most Local Authorities showed willingness to share data, however this tended to happen on an informal 'when requested' basis. Some Local Authorities reported sharing data with other Local Authorities more formally through obesity network meetings or emails, but this was on a regional rather than national level. Suggestions for future sharing mainly focused on developing online networks, forums or webinars which would enable data to be accessed both on a regional and national level.

- **INT 13:** *'I know in the sexual health areas they have like a forum or something, a website and they all sort of meet up and share best practice and they can ask questions online and things like that, so something like that for weight management would be good'*
- **INT 14:** *'I think there could be like a national monitoring …………………It would be useful to be able to know exactly what data is needed and have methods for having that all collected in one place by one system and then to be able to pull reports from that system locally, regionally, sub-regionally, nationally and even if we could go down to a very local level even a ward level'.*

Some Local Authorities felt that regional and national child obesity commissioner meetings would be useful. A few barriers mentioned to sharing data included time pressures, the commercially sensitive nature of some information and potential competition between Local Authorities, though most did not feel that the latter was a significant issue.

For peer review only - http://bmjopen.bmj.com/site/about/guidelines.xhtml

Within Local Authorities, evaluation or performance management data was mainly used to reshape and improve services and sometimes to promote the service and secure future funding.

Future Directions

1. Guidance needed on service specifications and contracts

Many Local Authorities commented on the lack of consistency in service provider contracts, specifications and outcomes measured across different Local Authorities. They felt that detailed practical guidance with sample service specifications and service provider contracts would be useful, including detailed guidance on what exactly the service should be aiming for in terms of weight loss and other objectives.

- **INT 19**: *I mean there's no like commissioning guidance on weight management programmes you know if that appears on my desk I'd be a very happy bunny because you know then it will tell me exactly what I need to look for, exactly what needs to be achieved. But we don't have a guidance that tells us that you know this is what you should expect from your provider.*
- **INT 17**: *I know trying to find some sort of consistency I think from a contracts point of view, it's been helpful that in other services, not children's weight management where we have had collaborative working around specifications and contracts and then obviously their local detail has been added to it.*

2. Cost Benefit Analyses Tool

In the current economic climate, a few Local Authorities suggested that it would be helpful if researchers developed a cost-benefit analysis tool which they could use for their child weight management programmes to justify allocation of money to these programmes.

- **INT 18:** *'a cost analysis tool. So, in terms of if X loses 5% in terms of weight loss, what that saves NHS/CCG/whoever it may be long term, because we have these cost analysis tools for *another service within the LA*, we have GP cost per hour, things like that, but we don't have anything for weight management for young people, but a cost analysis tool would be great'.*

For peer review only - http://bmjopen.bmj.com/site/about/guidelines.xhtml

Discussion

Main Findings

Data from the online survey demonstrated no Local Authorities were aware of any evidence published in peer-reviewed journals supporting the effectiveness of their specific tier 2 weight management service at improving BMI centile. Service evaluations were not consistently conducted. There was little consistency in methods for reporting change in weight status. The mean cost of the service per child completing the intervention was £1,312 and the mean actual reach of the service (i.e. children completing the intervention) to overweight or obese children within a LA was only 1.2%.

The qualitative research revealed the complexities of 'in-house' contracts in some Local Authorities. There were similarities between Local Authorities in the length of the intervention programme commissioned, the timing of data collection points and the outcomes measured. There were inconsistencies in the tools used to measure these outcomes which complicates meaningful comparisons of data between Local Authorities. Formal sharing of data between Local Authorities was lacking. Local Authorities identified many challenges facing their service including difficulties in collecting long-term data, lack of validated tools, the questionable reliability of self-report data, lack of engagement with the service and lack of resources especially in the current financial climate.

Many Local Authorities described the pressure on their service to reduce the prevalence of obesity but felt that a 'whole systems approach' was needed to tackle this problem rather than over-reliance on a single service.

Some Local Authorities felt more detailed guidance was needed on service specifications and contracts. Development of a cost-benefit analysis tool was also discussed by a few Local Authorities.

Meaning of the Findings: Implications for policy makers and clinicians

There is currently no way of easily comparing BMI z-score or other outcome data between different tier 2 weight management programmes across multiple Local Authorities in England. Although PHE have recently developed data entry forms, there is no mandatory system in place requiring Local Authorities to submit this information so it can be collated onto a central database for analysis [16]. Where data are shared, this is usually done on an informal basis at a local level. This is surprising given that the online survey highlighted that no Local Authorities knew of any peer-reviewed evidence supporting the effectiveness of their service at influencing weight status. In addition, there are very few UK-based research trials demonstrating a clinically significant reduction in BMI z-score in school aged children (defined as mean BMI SDS reduction of ≥ 0.25) [17 18 19]. A recent systematic review by Burchett et al reported only five of the thirty interventions included in the review reduced BMI z-score by ≥ 0.25 [18]. Of these five interventions, none were conducted in the UK and only 1 involved children of school-age.

For peer review only - http://bmjopen.bmj.com/site/about/guidelines.xhtml

Given the current economic climate and lack of evidence regarding long-term effectiveness of these interventions, it would seem wise to ensure that outcome data were being collected in a standardised format and that these data were compared and shared. This could help local and national agencies such as Public Health England to make evidence-based cost-effective commissioning decisions as the data in this paper suggests that these decisions are currently being conducted without good quality evidence. However, even if this was achieved, many Local Authorities have already alluded to the difficulties in collecting long-term data and so it is likely that there would be gaps in these data. It is also plausible that where long term data are collected, no long-term effectiveness is demonstrated. This is possible given that the NICE evidence review supporting the PH47 guideline reported no statistically significant mean difference in BMI z-score in the long-term for lifestyle weight management interventions for children [15].

Many Local Authorities discussed the pressures on their service to reduce the prevalence of obesity. However, the actual mean reach of a service (i.e. children completing the intervention) to overweight or obese children within a LA was 1.2%. It is therefore unrealistic to expect these services to influence obesity prevalence rates. Population measures are needed to have population level effects and it is therefore unclear where Tier 2 services such as those evaluated fall within the overall obesity strategy as they are neither population focussed nor have a strong evidence base for clinically defined groups. Even if the service had the capacity to take a large proportion of the overweight or obese population, the programme still probably would not reach most of this population due to the difficulties in engagement discussed by Local Authorities in the telephone interviews. Problems engaging families with services have been recognised in the literature [21]. Many Local Authorities described the need for a 'whole-systems approach' to effectively tackle the problem of childhood obesity.

A whole systems approach recognises the need to address a complex multi-causal problem using multiple different approaches rather than through a single intervention alone [21 22 23]. On a Local Authority level, this may involve influencing and linking multiple sectors such as planning, housing and transport, to effect population level changes [22 23]. Public Health England have commissioned Leeds Beckett University to identify ways in which Local Authorities might achieve a successful whole systems approach [23].

Weaknesses

The sample size for the online survey and telephone interviews were relatively small, but there was good geographical representation across England and saturation was felt to have been achieved in the telephone interviews. It is also not mandatory for Local Authorities to commission a tier 2 weight management service, so some Local Authorities may have felt this research was irrelevant.

Although a topic guide was used for the interviews, further discussions were guided by the participant. This had the strength of allowing inductive analyses to be conducted but the weakness that the opinions of every interview participant on each of the themes reported may not have been captured. It is also important to note that the current study focused on

For peer review only - http://bmjopen.bmj.com/site/about/guidelines.xhtml

Local Authorities in England. This means that the generalisation of results to the rest of the UK and wider is unclear.

Finally, Local Authorities did not provide answers in a comparable format for all questions on the online survey. This was likely in part due to variation in the type and format of data collected by each LA. A recent PHE study also recognised this problem, reporting that the average change in BMI centile post programme and at 12 months could not be determined due to the heterogeneity of respondents [24]. To gain a true oversight of the effectiveness of lifestyle weight management programmes currently commissioned in the UK, there needs to be consistency in the outcomes measured and clear guidelines on what clinically significant outcomes these services should be aiming to achieve.

Strengths and Contextualisation

In 2015, PHE conducted a national mapping study of tier 2 and tier 3 weight management services [24]. The evidence-base for the commissioned service was determined by asking Local Authorities if they followed NICE guidance or not, rather than asking whether their commissioned service had evidence supporting effectiveness in the peer-reviewed literature, as in this study. This is an important distinction as using guidelines to facilitate commissioning decisions is different to demonstrating the effectiveness of a commissioned service, especially given the limitations of the evidence supporting the NICE PH47 guidelines.

The PHE mapping study stated that the most frequently reported cost per participant of the service was ≥£401 though there is no further breakdown on figures above this range nor any standard deviations or mean costs provided. As a result, it is not possible to estimate the cost-effectiveness of these interventions. Furthermore, the survey asks for the 'average cost of the intervention per participant' but does not specify whether this should be per participant referred, per participant starting the intervention or per participant completing the intervention [25]. The strengths of our study are that we distinguish costs between these groups and report their means (with standard deviations).

In order to determine whether participants are followed up long-term, the PHE mapping study asked 'How long are the providers required to follow up the participants?' The study then reported that 67% of services reported follow-up of participants for 12 months or more. However, being 'required' to follow-up doesn't mean that the data were collected for all these participants. The qualitative part of our study provided insight into the difficulties in collecting long-term data even when the specification to do so is present.

The qualitative aspect of the PHE study had some similarities with our research, reporting lack of evidence of long-term effectiveness, lack of validated tools, lack of clear guidance on specifications, lack of funding, lack of expertise and difficulties with recruitment.

Future Directions

Given the current financial climate and scarcity of resources, it may be more appropriate to invest in population measures rather than interventions that focus on a small proportion of the overweight and obese population with minimal evidence to justify the costs spent. In

For peer review only - http://bmjopen.bmj.com/site/about/guidelines.xhtml

Mexico, the tax on sugar sweetened beverages (SSBs) in 2013 was associated with fewer taxed beverages being bought and more untaxed beverages being bought [26]. A similar tax in California reduced SSB consumption in low-income neighbourhoods [27]. Other strategies including reduction of TV advertising of high fat and/or high sugar foods and drinks to children have proven successful [28]. The WHO have also outlined a number of other effective population-based measures for preventing childhood obesity which include nutritional labelling of foods, policies aimed at the marketing of unhealthy foods and drinks, food taxes and subsidies, transport policies, increasing space for recreational activity and multi-component mass media campaigns.

In the present format, tier 2 weight management services for overweight and obese children are very unlikely to have any impact on the prevalence of childhood obesity and peer-reviewed evidence of any long-term benefits even within the small numbers of children reached by these services, is weak. If these services are to be continued, clear thought needs to be given to the goals of the service and a more robust system needs to be developed to determine whether these goals are being met, whether the service is cost-effective and if it is the best use of limited resources in the current economic climate.

Conclusion

Our results show that none of the participating Local Authorities were aware of any peer-reviewed evidence supporting the effectiveness of their specific commissioned service. Despite this, there was no national formal sharing of data to enable oversight of the efficacy of commissioned services across Local Authorities in England to help inform future commissioning decisions. Challenges with long-term data collection, service engagement, funding and the pressure to reduce the prevalence of obesity were frequently mentioned. The need for a 'whole-systems approach' to effectively tackle obesity was discussed. In the future, consideration needs to be given as to whether evidence-based, population-level interventions should be given preferential funding rather than small-scale lifestyle weight management services with uncertainty regarding long-term effectiveness.

For peer review only

For peer review only - http://bmjopen.bmj.com/site/about/guidelines.xhtml

Contributors and Guarantor: JHS, RJ, DS, RK and RM conceived and designed the study. Provisional analyses of the online survey data were conducted by RM and the telephone interview data by RM and AS. The results and analyses were reviewed and discussed by JHS, RJ and DS. RM drafted the manuscript. All authors revised and approved the final manuscript. The corresponding author attests that all listed authors meet authorship criteria and that no others meeting the criteria have been omitted. RM is guarantor.

Acknowledgements: RM is funded by the NIHR (NIHR ACF 2014-2017, NIHR In-Practice Fellowship 2018-2021). RJ is partly funded by National Institute for Health Research (NIHR) Collaboration for Leadership in Applied Health Research and Care West (CLAHRC West) at University Hospitals Bristol NHS Foundation Trust. RK works in the Centre for the Development and Evaluation of Complex Interventions for Public Health Improvement (DECIPHer), a UKCRC Public Health Research Centre of Excellence: joint funding (MR/KO232331/1) from the British Heart Foundation, Cancer Research UK, Economic and Social Research Council, Medical Research Council, the Welsh Government and the Wellcome Trust, under the auspices of the UK Clinical Research Collaboration, is gratefully acknowledged. J.H-S. is supported by the National Institute of Health Research (NIHR) Biomedical Research Centre at the University Hospitals Bristol National Health Service (NHS) Foundation Trust and the University of Bristol, Bristol, UK. DS works in in CAPC PHS funded by HEE Severn.

Disclaimer: The views expressed in this publication are those of the authors and not necessarily those of the NHS, the National Institute for Health Research or the Department of Health and Social Care.

Copyright and Licence: The Corresponding Author has the right to grant on behalf of all authors and does grant on behalf of all authors, a worldwide licence (http://www.bmj.com/sites/default/files/BMJ%20Author%20Licence%20March%202013.doc) to the Publishers and its licensees in perpetuity, in all forms, formats and media (whether known now or created in the future), to i) publish, reproduce, distribute, display and store the Contribution, ii) translate the Contribution into other languages, create adaptations, reprints, include within collections and create summaries, extracts and/or, abstracts of the Contribution and convert or allow conversion into any format including without limitation audio, iii) create any other derivative work(s) based in whole or part on the on the Contribution, iv) to exploit all subsidiary rights to exploit all subsidiary rights that currently exist or as may exist in the future in the Contribution, v) the inclusion of electronic links from the Contribution to third party material where-ever it may be located; and, vi) licence any third party to do any or all of the above. All research articles will be made available on an open access basis (with authors being asked to pay an open access fee—see http://www.bmj.com/about-bmj/resources-authors/forms-policies-and-checklists/copyright-open-access-and-permission-reuse). The terms of such open access shall be governed by a Creative Commons licence—details as to which Creative Commons licence will apply to the research article are set out in our worldwide licence referred to above.

For peer review only

Competing Interest Statement*:* All authors have completed the ICMJE uniform disclosure form at www.icmje.org/coi_disclosure.pdf. During the conduct of this study, Dr. Mears and Professor Hamilton-Shield reports grants from National Institute of Healthcare Research, Professor Jago reports grants from NIHR - CLARHC West and Ms. Kipping reports grants from DECIPHer, grant funding from a collection of charities and funding councils as outlined in the acknowledgments section of this paper. Professor Sharp and Ms Sharma have nothing to disclose.

Patient and Public Participation: Patients/public were not involved in the design of this study or to interpret results.

Data Sharing Statement: Participants gave consent for collection of data which would then be anonymised, so it is not be possible to share individual Local Authority data.

For peer review only

For peer review only - http://bmjopen.bmj.com/site/about/guidelines.xhtml

References

1. Collaboration NCDRF. Worldwide trends in body-mass index, underweight, overweight, and obesity from 1975 to 2016: a pooled analysis of 2416 population-based measurement studies in 128.9 million children, adolescents, and adults. *Lancet* 2017;390(10113):2627-42.
2. World Health Organisation (WHO). Tenfold increase in childhood and adolescent obesity in four decades: new study by Imperial College London and WHO http://www.who.int/mediacentre/news/releases/2017/increase-childhood-obesity/en/2017
3. World Health Organisation (WHO). Global Health Observatory Data, 2016 http://www.who.int/gho/ncd/risk_factors/overweight_obesity/overweight_adolescents/en/
4. Singh AS, Mulder C, Twisk JW, et al. Tracking of childhood overweight into adulthood: a systematic review of the literature. *Obesity reviews : an official journal of the International Association for the Study of Obesity* 2008;9(5):474-88. doi: 10.1111/j.1467-789X.2008.00475.x [published Online First: 2008/03/12]
5. Finkelstein EA, Graham WC, Malhotra R. Lifetime direct medical costs of childhood obesity. *Pediatrics* 2014;133(5):854-62.
6. NICE. Weight management: lifestyle services for overweight or obese children and young people. PH47. 2013. https://www.nice.org.uk/guidance/ph47/history
7. NHS England. Report of the working group into: Joined up clinical pathways for obesity, 2014. https://www.england.nhs.uk/wp-content/uploads/2014/03/owg-join-clinc-path.pdf:
8. National Childhood Measurement Programme England 2014-2015: Tables. NHS Digital. https://digital.nhs.uk/catalogue/PUB19109
9. Public Health England. Weight management: guidance for commissioners and providers. 2017. https://www.gov.uk/government/collections/weight-management-guidance-for-commissioners-and-providers.
10. Ford AL, Hunt LP, Cooper A, et al. What reduction in BMI SDS is required in obese adolescents to improve body composition and cardiometabolic health? *Archives of Disease in Childhood*;95(4):256-61.
11. Reinehr T, Lass N, Toschke C, et al. Which Amount of BMI-SDS Reduction Is Necessary to Improve Cardiovascular Risk Factors in Overweight Children? *Journal of Clinical Endocrinology & Metabolism*;101(8):3171-9.
12. Peirson L, Fitzpatrick-Lewis D, Morrison K, et al. Treatment of overweight and obesity in children and youth: a systematic review and meta-analysis. *CMAJ open*;3(1):E35-46.
13. Seburg EM, Olson-Bullis BA, Bredeson DM, et al. A Review of Primary Care-Based Childhood Obesity Prevention and Treatment Interventions. *Current Obesity Reports*;4(2):157-73.
14. Upton P, Taylor C, Erol R, et al. Family-based childhood obesity interventions in the UK: a systematic review of published studies. *Community Practitioner*;87(5):25-9.
15. F. Morgan AW, S. Whitehead, S. Brophy, H. Morgan, R. Turley, R. Kipping, J. White, W. Hollingworth Managing overweight and obesity among children and young people: lifestyle weight management services. Review 1: Effectiveness and cost effectiveness of lifestyle weight management services for children and young people. *NICE* 2013

For peer review only - http://bmjopen.bmj.com/site/about/guidelines.xhtml

16. Public Health England. Child weight management services: collect and record data. Capturing data: a tool to collect and record child weight management services data, 2017. https://www.gov.uk/government/publications/child-weight-management-services-collect-and-record-data
17. Al-Khudairy L, Loveman E, Colquitt JL, et al. Diet, physical activity and behavioural interventions for the treatment of overweight or obese adolescents aged 12 to 17 years. *Cochrane Database of Systematic Reviews*;6:CD012691.
18. Burchett HED, Sutcliffe K, Melendez-Torres GJ, et al. Lifestyle weight management programmes for children: A systematic review using Qualitative Comparative Analysis to identify critical pathways to effectiveness. *Preventive Medicine*;106:1-12.
19. Mead E, Brown T, Rees K, et al. Diet, physical activity and behavioural interventions for the treatment of overweight or obese children from the age of 6 to 11 years. *Cochrane Database of Systematic Reviews*;6:CD012651.
20. Kelleher E, Davoren MP, Harrington JM, et al. Barriers and facilitators to initial and continued attendance at community-based lifestyle programmes among families of overweight and obese children: a systematic review. *Obesity Reviews*;18(2):183-94.
21. Lee BY, Bartsch SM, Mui Y, et al. A systems approach to obesity. *Nutrition Reviews*;75(suppl 1):94-106.
22. Rutter H, Savona N, Glonti K, et al. The need for a complex systems model of evidence for public health. *Lancet*;390(10112):2602-04.
23. Leeds Beckett University. Whole Systems Approach to Tackle Obesity, 2018. http://www.leedsbeckett.ac.uk/wholesystemsobesity/
24.Public Health England. National Mapping of Weight Management Services, 2015. https://assets.publishing.service.gov.uk/government/uploads/system/uploads/attachment_data/file/484115/Final_Weight_Management_Mapping_Report.pdf
25.Public Health England. National mapping of weight management services: e-survey and face to-face approach, 2015. https://assets.publishing.service.gov.uk/government/uploads/system/uploads/attachment_data/file/484068/National_mapping_of_weight_management_services_Annex_2.pdf
26. Colchero MA, Popkin BM, Rivera JA, et al. Beverage purchases from stores in Mexico under the excise tax on sugar sweetened beverages: observational study. *BMJ*;352:h6704.
27. Falbe J, Thompson HR, Becker CM, et al. Impact of the Berkeley Excise Tax on Sugar-Sweetened Beverage Consumption. *American Journal of Public Health*;106(10):1865-71.
28. Haby MM, Vos T, Carter R, et al. A new approach to assessing the health benefit from obesity interventions in children and adolescents: the assessing cost-effectiveness in obesity project. *International Journal of Obesity*;30(10):1463-75.

For peer review only - http://bmjopen.bmj.com/site/about/guidelines.xhtml

University of BRISTOL

Supplementary File 1 – Online Survey Questions:

National Survey of LA Commissioned Weight Management Services for Overweight / Obese Children in 2014/15

	**Informed Consent for Online Survey**
	In order to take part in this online survey, we need to obtain your informed consent. Please read the following five statements carefully.<br>1. I confirm that I have read and understood the participant information.<br>2. I am willing to take part in the survey<br>3. I understand that my name will be kept anonymous however due to the nature of the study, it is not possible to anonymise the local authority name<br>4. I understand that information collected (name of survey participant anonymised but name of local authority not anonymised) will be stored for 10 years in data sets within a secure facility in accordance with the Data Protection Act 1998 and this data may be used in publications or presentations to relevant audiences or shared with other researchers.<br>5. I understand that I can withdraw from the study at any point prior to March 21st 2016 by emailing Dr Ruth Mears on rm14101@bristol.ac.uk
**Question 1**	Please confirm that you have read, understood and agree to the above five statements
**Response 1**	
	**Participant & Local Authority Details**
**Question 2**	What local authority do you work in?<br>*Please note that if you work for multiple local authorities, you will need to fill out a new survey for each local authority that you work for.*
**Response 2**	
	**Tier 2 Weight Management Services for Overweight/Obese Children in your Local Authority**
**Question 3**	Please name a Tier 2 weight management service commissioned by your local authority for overweight / obese children aged 4-16 years during the financial year April 2014-March 2015?
**Response 3**	
**The following questions relate to the Tier 2 weight management service you have named above.**	
**Question 4**	What evidence are you aware of regarding the effectiveness of the service commissioned between April 2014 – March 2015 at reducing BMI centile / BMI % / BMI z-score or BMI? Please choose (highlight) from the below list;
**Response 4**	– *Data published in a peer reviewed journal – independently collected (i.e. data collected by a person who is NOT an employee of the weight management service provider)*

For peer review only

University of BRISTOL

	– *Data published in a peer reviewed journal – internally collected (i.e. data collected by a person who IS an employee of the weight management service provider)*<br>– *Published in an alternative source – independently collected (i.e. data collected by a person who is NOT an employee of the weight management service provider)*<br>– *Published in an alternative source – internally collected (i.e. data collected by a person who IS an employee of the weight management service provider)*<br>– *Unpublished data*<br>– *Other*<br>– *No evidence*
**Question 5**	Please specify where the evidence can be found regarding the effectiveness of the services commissioned during the year April 2014-March 2015 at reducing BMI centile / BMI % / BMI z-score or BMI?<br>*(e.g. publication details / website address etc. If the data is unpublished, please email details to rm14101@bristol.ac.uk)*
**Response 5**	
**Question 6**	Since the service was commissioned (i.e. contract start date), had it been evaluated within your local authority?
**Response 6**	
**Question 7**	As part of the service evaluation, was change in weight status measured (e.g. change in BMI, BMI%, BMI centile or BMI z-score?)
**Response 7**	
**Question 8**	If change in weight status was measured, what were the results?<br>*Please write the time frame in which this change occurred e.g. Reduction of BMI centile by 0.9% (SD) over 1 year (2014/15). If this is available for different age groups, please indicate the results by age group.*
**Response 8**	
**Question 9**	What was the total cost of the service for the local authority between April 2014 to March 2015?<br>*If data cannot be provided please specify the time period and costs in the format you have available e.g. cost per child per course in August 2014.*
**Response 9**	
**Question 10**	What was the maximum number of participants that could have been accommodated by the commissioned service between April 2014 to March 2015?<br>*Where possible, please provide data on maximum commissioned capacity for a one year time frame from 2014 to 2015. If this data cannot be provided, please specify the time period and maximum capacity of the service in the format you have available e.g. maximum capacity of 60 children per course in 2014, total of 10 courses in 2014.*

For peer review only

**Response 10**	
**Question 11**	How many children were referred to the service between April 2014 to March 2015?<br>*If this data cannot be provided please specify the time period and referral data in the format you have available*
**Response 11**	
**Question 12**	How many children completed the intervention between April 2014 to March 2015?<br>*If data cannot be provided please specify the time period and number completing the intervention in the format you have available*
**Response 12**	
	**Thank you for taking the time to complete this survey. Please consider taking part in the second phase of our research.**
**Question 13**	Thank you for taking the time to complete this survey.<br><br>Would you like to receive a summary of the results and analysis by email?<br><br>If you answered yes to the above question, please provide us with your email address
**Response 13**	
**Question 14**	If you answered yes to the above question, please provide us with your email address
**Response 14**	
	**Telephone Interview**

The second phase of our research will involve a telephone interview exploring commissioners' views and experiences in the evaluation of weight management services for overweight and obese children. There is little qualitative evidence available regarding service evaluation data collected by commissioners and this research aims to fill the gap in the literature. We will explore the views of commissioners on the role and value of service evaluation data, the barriers and facilitators to collecting and processing this data and finally how to ensure evaluation data is useful. Performance management of services will also be explored.

If you are interested in participating in the interview, please can you provide your name and contact details (email and/or telephone number) below. We will then email you a participant information sheet providing further details about what the interview involves. After reading the information sheet, if you decide you would like to take part, you will need to fill out the consent form and send it to rm14101@bristol.ac.uk. Dr Ruth Mears will then contact you to arrange a convenient time for you to conduct the telephone interview

……
……
……

For peer review only

For peer review only

COREQ (COnsolidated criteria for REporting Qualitative research) Checklist

A checklist of items that should be included in reports of qualitative research. You must report the page number in your manuscript where you consider each of the items listed in this checklist. If you have not included this information, either revise your manuscript accordingly before submitting or note N/A.

Topic	Item No.	Guide Questions/Description	Reported on Page No.
**Domain 1: Research team and reflexivity**			
Personal characteristics			
Interviewer/facilitator	1	Which author/s conducted the interview or focus group?	Pg 3
Credentials	2	What were the researcher's credentials? E.g. PhD, MD	MBBCh, BSc
Occupation	3	What was their occupation at the time of the study?	GP Registrar, NIHR ACF
Gender	4	Was the researcher male or female?	Female
Experience and training	5	What experience or training did the researcher have?	MBBCh, BSc
Relationship with participants			
Relationship established	6	Was a relationship established prior to study commencement?	No
Participant knowledge of the interviewer	7	What did the participants know about the researcher? e.g. personal goals, reasons for doing the research	RM working part-time in general practice and part-time in research
Interviewer characteristics	8	What characteristics were reported about the inter viewer/facilitator? e.g. Bias, assumptions, reasons and interests in the research topic	As above
**Domain 2: Study design**			
Theoretical framework			
Methodological orientation and Theory	9	What methodological orientation was stated to underpin the study? e.g. grounded theory, discourse analysis, ethnography, phenomenology, content analysis	Pg 4, Inductive thematic analysis
Participant selection			
Sampling	10	How were participants selected? e.g. purposive, convenience, consecutive, snowball	Pg 3
Method of approach	11	How were participants approached? e.g. face-to-face, telephone, mail, email	Pg 5
Sample size	12	How many participants were in the study?	Pg 6
Non-participation	13	How many people refused to participate or dropped out? Reasons?	From the online survey, 31 expressed an interest to participate in the telephone interviews.

Setting			
Setting of data collection	14	Where was the data collected? e.g. home, clinic, workplace	Workplace
Presence of nonparticipants	15	Was anyone else present besides the participants and researchers?	Nil with RM. Interview participants may be sharing an office.
Description of sample	16	What are the important characteristics of the sample? e.g. demographic data, date	Pg 6
Data collection			
Interview guide	17	Were questions, prompts, guides provided by the authors? Was it pilot tested?	Pg 3
Repeat interviews	18	Were repeat inter views carried out? If yes, how many?	No
Audio/visual recording	19	Did the research use audio or visual recording to collect the data?	Pg 4
Field notes	20	Were field notes made during and/or after the inter view or focus group?	No
Duration	21	What was the duration of the inter views or focus group?	Pg 6
Data saturation	22	Was data saturation discussed?	Pg 4
Transcripts returned	23	Were transcripts returned to participants for comment and/or correction?	No
**Topic**	**Item No.**	**Guide Questions/Description**	**Reported on Page No.**
**Domain 3: analysis and findings**			
Data analysis			
Number of data coders	24	How many data coders coded the data?	Pg 4
Description of the coding tree	25	Did authors provide a description of the coding tree?	No
Derivation of themes	26	Were themes identified in advance or derived from the data?	Pg 4
Software	27	What software, if applicable, was used to manage the data?	Pg 4
Participant checking	28	Did participants provide feedback on the findings?	Not yet. Summary will be sent to participants in due course.
Reporting			
Quotations presented	29	Were participant quotations presented to illustrate the themes/findings? Was each quotation identified? e.g. participant number	Pg 6-13
Data and findings consistent	30	Was there consistency between the data presented and the findings?	Pg 14-17
Clarity of major themes	31	Were major themes clearly presented in the findings?	Pg 6-13
Clarity of minor themes	32	Is there a description of diverse cases or discussion of minor themes?	Pg 6-13

Developed from: Tong A, Sainsbury P, Craig J. Consolidated criteria for reporting qualitative research (COREQ): a 32-item checklist for interviews and focus groups. *International Journal for Quality in Health Care*. 2007. Volume 19, Number 6: pp. 349 – 357

For peer review only

Once you have completed this checklist, please save a copy and upload it as part of your submission. DO NOT include this checklist as part of the main manuscript document. It must be uploaded as a separate file.

For peer review only

BMJ Open

A mixed methodology study exploring how lifestyle weight management programmes for children are commissioned and evaluated in England.

Journal:	*BMJ Open*
Manuscript ID	bmjopen-2018-025423.R1
Article Type:	Research
Date Submitted by the Author:	05-May-2019
Complete List of Authors:	Mears, Ruth; Centre for Academic Primary Care, Bristol Medical School, University of Bristol,; Centre for Exercise Nutrition and Health Sciences, School for Policy Studies, University of Bristol,<br>Jago, Russ; Centre for Exercise Nutrition and Health Sciences, School for Policy Studies, University of Bristol,<br>Sharp, Deborah; Centre for Academic Primary Care, Bristol Medical School, University of Bristol,<br>Patel, Anamica; Centre for Exercise Nutrition and Health Sciences, School for Policy Studies, University of Bristol<br>Kipping, Ruth; Department of Population Health Sciences, Bristol Medical School, University of Bristol,; Centre for the Development and Evaluation of Complex Interventions for Public Health Improvement (DECIPHer),<br>Shield, Julian; NIHR Bristol Biomedical Research Centre, Nutrition Theme, University of Bristol,; Faculty of Health Sciences, University of Bristol, BS2 8DZ, UK
**Primary Subject Heading**:	Public health
Secondary Subject Heading:	Evidence based practice
Keywords:	obesity, lifestyle weight management services, children, commissioning, evaluation

SCHOLARONE™
Manuscripts

A mixed methodology study exploring how lifestyle weight management programmes for children are commissioned and evaluated in England

Mears R [1,2], Jago R [2], Sharp D [1], Patel A[2], Kipping R [3,4], Shield JPH [5,6]

Centre for Academic Primary Care, Bristol Medical School, University of Bristol, BS8 2PS, UK [1]. Centre for Exercise Nutrition and Health Sciences, School for Policy Studies, University of Bristol, BS8 1TZ, UK [2]. Department of Population Health Sciences, Bristol Medical School, University of Bristol, BS8 2PS, UK [3]. Centre for the Development and Evaluation of Complex Interventions for Public Health Improvement (DECIPHer), Bristol, BS8 2PS, UK [4]. NIHR Bristol Biomedical Research Centre, Nutrition Theme, University of Bristol, BS1 2NT, UK [5]. Faculty of Health Sciences, University of Bristol, BS2 8DZ, UK [6].

Dr Ruth Mears (Corresponding Author)
Dr Ruth Mears. Centre for Exercise Nutrition and Health Sciences, School for Policy Studies, University of Bristol, BS8 2PS. rm14101@bristol.ac.uk Telephone: +44 (0)117 954 6755
Fax: +44(0)117 954 6756

Author Positions: Dr Ruth Mears – NIHR Academic Clinical Fellow in Primary Care. Professor Russell Jago - Professor of Paediatric Physical Activity and Public Health. Professor Debbie Sharp - Professor of Primary Health Care. Ms. Anamica Patel - MSc Student. Ms. Ruth Kipping - Consultant Senior Lecturer in Public Health. Professor Hamilton-Shield - Professor in Diabetes and Metabolic Endocrinology.

For peer review only

For peer review only - http://bmjopen.bmj.com/site/about/guidelines.xhtml

Abstract

Objectives: To assess how lifestyle weight management programmes for children aged 4-16 years in England are commissioned and evaluated at the local level.

Design: This was a mixed methods study comprising an online survey and semi-structured telephone interviews.

Setting: An online survey was sent to all Local Authorities (LAs) in England regarding lifestyle weight management services commissioned for children aged 4-16 years. Online survey data were collected between February and May 2016 and based on services commissioned between April 2014 and March 2015. Semi-structured telephone interviews with LA staff across England were conducted between April and June 2016.

Participants: Commissioners or service providers working within the Public Health Department of LAs.

Main outcome measures: The online survey collected information on the evidence-base, costs, reach, service usage and evaluation of child lifestyle weight management services. The telephone interviews explored the nature of child weight management contracts commissioned by LAs, the type of outcome data collected and whether these data were shared with other LAs or organisations, the challenges faced by these services and the perceived 'markers of success' for a programme.

Results: The online survey showed that none of the participating LAs were aware of any peer-reviewed evidence supporting the effectiveness of their specific commissioned service. Despite this, the telephone interviews revealed that there was no national formal sharing of data to enable oversight of the effectiveness of commissioned services across LAs in England to help inform future commissioning decisions. Challenges with long-term data collection, service engagement, funding and the pressure to reduce the prevalence of obesity were frequently mentioned.

Conclusions: Robust independent, cost-effectiveness analyses of obesity strategies are needed to determine the appropriate allocation of funding to lifestyle weight management treatment services, population-level preventative approaches or development of whole-system approaches by an LA.

Strengths and limitations of this study

- There has been no previous independent, peer-reviewed research study assessing how lifestyle weight management programmes in childhood are being commissioned and evaluated across Local Authorities (LAs) in England.
- The response rate for the online survey was lower than desired however there was good geographical representation across England.
- The current study focused on LAs in England so generalisation of results to the rest of the UK and wider is unclear.
- The change in weight status and cost data provided by LAs precluded meaningful statistical analyses so it is impossible to comment on the cost-effectiveness of, or between, commissioned services.

For peer review only - http://bmjopen.bmj.com/site/about/guidelines.xhtml

- There were no freedom of information requests submitted to LAs who did not complete the online survey and it is possible further data could have been obtained through this route.

Introduction

In the past four decades, there has been a tenfold increase in the number of obese children and adolescents worldwide [1 2]. In the United Kingdom, 31.1% of children and adolescents were classified as overweight or obese in 2016 [3]. These children and adolescents are more likely to become overweight or obese adults and suffer health related consequences [4]. This presents a major public health challenge [5]. In the UK, weight management strategies are classified into tier 1 (those that focus on preventing obesity), tier 2 (lifestyle weight management services), tier 3 (specialist obesity services) and tier 4 services (pharmacological or surgical treatments for obesity) [6]. Tier 1 and 2 services are commissioned by public health departments working within Local Authorities (LAs). Tier 3 and 4 services are commissioned by a combination of clinical commissioning groups (CCG's) and NHS England[7]. CCG's are responsible for the planning and commissioning of health care services for their local area and are assured by NHS England [8]. In 2013, Public Health England (PHE) was formed as a separate entity to NHS England as public health care transitioned from the NHS to LAs under the Health and Social Care Act 2012 [9].

This paper focuses on tier 2 weight management services commissioned by LAs across England for school-aged children (aged 4-16 years). There are 152 LAs in England [10] and each LA may choose to commission services from a different tier 2 service provider. Although there is guidance from the National Institute of Clinical Excellence (NICE) and PHE regarding what these services should comprise [6 11], the specific weight management programmes have rarely been independently evaluated and published. Furthermore, there are very few UK-based, randomised trials in the peer-reviewed literature demonstrating a clinically significant reduction in BMI z-score (defined as minimum BMI SDS reduction of ≥ 0.25)[12 13] through lifestyle weight management programmes alone for school aged-children [14 15 16]. Even the evidence reviews supporting the NICE public health guidance (PH47), only reported a post-intervention pooled reduction in BMI z-score of -0.17 (95% CI = -0.3 to -0.04, p=0.01) which was attenuated when long-term data (≥ 6 months) were used (Standardised Mean Difference (SMD) = -0.07; CI 95% = −0.15 to 0.02, p = 0.12) [17].

LAs usually monitor their tier 2 weight management services through 'Performance Management' meetings, although they may also conduct service evaluations. NICE recommends that monitoring focuses on sustaining long term changes [6], despite their evidence reviews showing little efficacy for these interventions in the long-term [17]. Given the poor evidence-base for tier 2 weight management services, it is important to understand more about the nature of the contracts commissioned by LAs, the monitoring of outcomes and the challenges facing these services. In addition, given the current financial climate in public health, with spending estimated by the King's Fund to be 8% lower four

For peer review only - http://bmjopen.bmj.com/site/about/guidelines.xhtml

years after public health moved from the NHS to LAs [18], it is important to explore whether these services are a good use of limited resources.

This mixed methods study uses quantitative methods (an online survey) to determine the evidence-base underpinning the local service provided, costs, reach, service usage and evaluation of tier 2 weight management programmes commissioned by LAs across England for children aged 4-16 years. Qualitative methods (semi-structured telephone interviews) explore the nature of childhood tier 2 weight management contracts commissioned by LAs, the type of outcome data collected and whether these data are shared, the challenges faced by these services and the perceived ‘markers of success’ for a programme. Finally, the data collected from both the online survey and telephone interviews examine whether lifestyle weight management programmes are a good use of limited resources.

For peer review only

For peer review only - http://bmjopen.bmj.com/site/about/guidelines.xhtml

Methods

Participants and Recruitment

A list of all 152 LAs in England was derived from 2014/2015 National Child Measurement Programme (NCMP) datasets [19]. The Director for Public Health for each LA was contacted by email and asked to identify the relevant person within their LA responsible for the commissioning of childhood tier 2 weight management services. An email was sent to this person asking if they would be willing to participate in an online survey exploring tier 2 weight management services for school-aged children commissioned between April 2014 and March 2015. If no response to the email was received, a further email was sent.

The final page of the online survey provided information about the second phase of the study (telephone interviews) and invited those interested in taking part to leave their contact details. In addition, some of those LAs who declined to participate in the online survey, were also invited by email to take part in the telephone interviews.

Design of Online Survey and Telephone Interview Guide

The online survey (Supplementary File 1) and interview guide (Supplementary File 2) were developed by RM, RJ, DS, JHS and RK. Development of the survey and interview guide were informed by the collective experiences of these clinicians and researchers in the field of childhood weight management and through addressing gaps in the current literature.

Ethical Approval

Ethical approval was granted by the School for Policy Studies Research Ethics Committee at the University of Bristol. Informed consent was obtained in written format for the online survey and in written or verbal format for the telephone interviews.

Online Survey

The online survey comprised 10 questions relating to tier 2 weight management services commissioned by the LA for overweight or obese children aged 4-16 years in March 2014 to April 2015. The survey collected data on the evidence-base supporting the commissioned intervention, the cost of the service, the maximum number of participants the service could have accommodated, the number of children referred, the number of children completing the intervention, whether a service evaluation had been conducted and the changes in weight status measured through service evaluation. Data were collected between February and May 2016 and analysed in Microsoft Excel.

Telephone Interviews

Semi-structured telephone interviews were conducted by RM between April and June 2016. The interview guide had a common framework but was adapted during the interview as guided by the participants' responses.

The interviews required participants to reflect on their experiences of tier 2 weight management services for school-aged children within their LA but was not confined to

For peer review only

experiences within the time-period specified in the online survey of March 2014-April 2015. This enabled a broader representation of experiences from interview participants. The interviews explored the nature of the contracts commissioned by LAs and the monitoring of these services through performance management and service evaluation. Specifically, the interviews explored whether outcome data were collected, whether these data were shared, the challenges identified through monitoring processes and the perceived 'markers of success' for the service.

Interviews were audio-recorded then transcribed verbatim by Bristol Transcription Service. All interview transcripts were anonymised by AP and uploaded to N-vivo 10.0 for inductive thematic analysis.

Data were organised into codes and themes and constantly revised and reviewed by two researchers working independently (RM and AP). Once coding was complete, both researchers discussed differences and links within and across themes before agreeing on the final themes. Themes were inductively and deductively elicited based on the interview guide and the information that emerged during the interviews. Data saturation was deemed to have been met when no new information emerged from the interviews which resulted in a sample of 20 participants [20].

Transparency statement

The online survey was conducted as originally planned. The telephone interviews initially aimed to explore service evaluation and performance management of tier 2 weight management services for children from a commissioner's perspective and experiences. As it emerged that some LAs run in-house contracts, participants were included who were within a LA but also service providers. The data which subsequently emerged focused the analysis on determining whether lifestyle weight management programmes were a good use of limited resources.

For peer review only

For peer review only - http://bmjopen.bmj.com/site/about/guidelines.xhtml

Results

Quantitative Data from Online Survey

Survey Respondents

Contact details for 103 LA ‘obesity leads’ were obtained through Directors of Public Health and via suggestions from PHE. Of these, 40 completed the survey, 24 declined to complete the survey and provided a reason (nil commissioned n=14, service decommissioned n=4, insufficient resources to complete the survey n=3, declined for other reasons n=3) and 39 did not complete the survey and did not provide a reason. Of the remaining 49 LAs, it is possible that the DPH forwarded our email onto the relevant contact but did not copy us in or that some of these LAs simply did not commission a tier 2 weight management service for children.

Geographical Location of Survey Respondents

The geographical location of the forty LAs who completed the online survey were; North West (n=10), North East (n=2), Yorkshire and the Humber (n=4), West Midlands (n=3), East Midlands (n=1), East of England (n=3), London (n=7), South West (n=7) and South East (n=3). The population of children aged 4-16 years within each of these forty LAs ranged from 16,000 to 186,000 (Mid-2014 Population Data from Office of National Statistics).

Evidence-base of Tier 2 Weight Management Service Commissioned

No LAs were aware of evidence published in peer-reviewed journals demonstrating that their service was effective at improving BMI centile (or other weight related measure). Service evaluations were conducted in 55% of LAs, of which 18% did not measure change in weight status as part of their service evaluation. Due to heterogeneity in the way in which outcome data for change in weight status were reported by LAs (e.g. proportion who reduced or maintained their BMI z-score, number who ‘lost weight’, % of children who reduced their BMI z-score by 3%, only 6 or 12 month data), it was not possible to make any meaningful interpretations or comparisons of these data.

Costs and Reach of the Service

Table 1 summarises the costs of the service. Some LAs were only able to provide estimates. Table 2 summarises the reach of services within an LA.

Table 1: Costs of the Service

	Mean cost (SD, n = number of LAs providing data)
Cost of the service per year to LA	£130,742 (SD £122,869, n=27)
Cost of the service per year per 10,000 children aged 4-16 years (of any weight) in LA	£29,397 (SD £30,003, n=27)

For peer review only - http://bmjopen.bmj.com/site/about/guidelines.xhtml

Cost of the service per overweight or obese child attending if maximum capacity of the service was reached	£558 (SD £408, n=18)
Cost of the service per child completing the intervention	£1,312 (SD £1342, n= 15)

Table 2: Reach of the Service

	Mean (SD, n)
Potential reach of the service (presuming maximum capacity was achieved) to overweight or obese children within a LA	3.5% (SD 6.9%, n=26) *
Estimated actual reach of the service (i.e. children completing the intervention) to overweight or obese children within a LA	1.2% (SD 1.6%, n=25) *

** These calculations used estimates of the prevalence of overweight or obese children within a LA aged 4-16 years (this was estimated using NCMP data from Reception and Year 6 and National Statistics population data for children aged 4-16 years).*

Qualitative Data from Telephone Interviews

Twenty telephone interviews were conducted with LAs (18 commissioners, 2 service providers within the LA – Interview number 18 and 20). Seventeen of the telephone interview participants had completed the online survey. Three had declined. The geographical location of the twenty LAs who completed the interview were; North West (n=8), North East (n=1), Yorkshire and the Humber (n=1), West Midlands (n=0), East Midlands (n=1), East of England (n=1), London (n=4), South West (n=3) and South East (n=1). Interviews were between 28 and 68 minutes in length.

Nature of Commissioning Contracts

Tier 2 weight management contracts were either between the LA and an external provider, or 'in-house' contracts (where the LA acts as both the commissioner and service provider). Some LAs reported running 'in-house' contracts as they could not afford to commission the service to an external provider. This was not a problem if the service was performing well, however if the service was underperforming, their options might be limited as they may not be able to go out to market due to financial and political pressures.

- **INT 3**: *'if they're not achieving their targets, they're not doing their job properly, so then we shouldn't be providing the service, but what is the alternative? It's too expensive to commission it out'*

One LA discussed the challenges of 'in-house' contracts from a leadership perspective, especially as their service was not meeting BMI targets.

For peer review only - http://bmjopen.bmj.com/site/about/guidelines.xhtml

- **INT 17:** *'To make it complicated our provider is also within the LA so there's a bit of – it's something that provides such a huge challenge just on its own because you've got provider senior leadership and commissioning senior leadership with different views'……… 'the service underachieved against the targets around BMI consistently over the last two years……..If they were an external provider it would probably be a different scenario'*

Outcome Data

All LAs collected outcome data through performance management processes and some also collected outcome data through service evaluation. Most interventions were around 12 weeks long with data collected at baseline and at the end of the intervention. Some LAs also attempted to collect longer-term data at 3 month, 6 months and/or 12 months. Although the general themes of data collected were similar (demographic data, retention, engagement, weight, self-esteem, confidence, behavioural change, physical activity, diet), the actual data were collected in different formats across some LAs. For example, some LAs measured physical activity via a seven-day recall questionnaire, others through a physical activity test and others by asking parents whether their children increased their activity levels or through assessing physical literacy.

Challenges identified through Service Evaluation and/or Performance Management Meetings

1. **Lack of long-term data**

Many participants mentioned the difficulties in collecting long-term follow-up data. This was attributed to a variety of factors including length of questionnaires, lack of parental confidence with the paperwork, too much effort for families to undertake, people moving around town, resource constraints of LA to capturing this data, lack of IT infrastructure and lack of engagement in both the intervention and the evaluation.

- **INT 14:** *'It becomes then quite time consuming to try and chase patients who engaged. People forget what they've done 12 months ago or more as well. …………it would be quite difficult with not having things like a GP surgeries infrastructure like EMIS where data gets held for years and years and it's there to use and accessed again'.*

2. **Lack of validated tools**

Some participants felt that there was a lack of validated tools to enable accurate outcome measures to be obtained.

- **INT 1:** *'We're looking for validated tools but there are just not that many great ones out there.'*

3. **Reliability of self-report data**

A few participants questioned the reliability of self-report data.

For peer review only - http://bmjopen.bmj.com/site/about/guidelines.xhtml

- **INT 3:** *'Other challenges are self-reporting. …. The physical activity and nutrition tend to be improved after ten weeks and sometimes you look on that a little cynically because the measurements haven't improved, so perhaps they're telling us what we want to hear, that can be a challenge'.*

4. **Lack of engagement**

Difficulties engaging children, parents and healthcare professionals with the service was mentioned by many of the LAs. This is summarised in Table 3.

Table 3: Challenges of Engaging Parents, Children and Health-Care Professionals with the service

Difficulties engaging parents	
Talking about the weight of a child can be highly emotive for parents;	**INT 19:** *'It's difficult with parents sometimes to explain to them that what they are doing at home is probably not the best thing for their child. That's quite difficult you know, that's their baby that's their child and they don't want to hear anything negative.'*
Parents often find it difficult to accept that their child is overweight;	**INT 5:** *'Parents often see their children as normal weight when they are in fact overweight and we know people often will refer to children who are a normal weight as a bit skinny,'*
Parents often do not recognise the role they need to play in engaging in the service as part of a 'family intervention' to improve their child's BMI centile	**INT 11:** *'So we say it has to be a family intervention. But they don't always see it that way. They just want the child to lose the weight and don't acknowledge their role in being the providers' food and the environment they grow up in'.*
Difficulties engaging children	
Engaging children with the service could be challenging;	**INT 2:** *'there is a lot of issues around recruitment and retentions with tier two services for children and also there's a great difficulty with actually the secondary aged children to get them sort of accessing services'.*
Difficulties engaging healthcare professionals	
Healthcare professionals can find it difficult to bring up weight status of a child with a parent.	**INT 4:** *'I think there's definitely issues there from what I've heard about professionals bringing things up with families'*

For peer review only - http://bmjopen.bmj.com/site/about/guidelines.xhtml

Some healthcare professionals fail to recognise overweight or obese children	**INT 5:** *'The GP will look at the child and say, it's just puppy fat, they'll grow out of it'.*<br>**INT 11:** *'We even get some out of school nurses say 'well, they're only just into the overweight category'. You know, the child is really athletic, they're really muscular'*
Lack of GP engagement	**INT 10:** *'GP's still struggle to engage with it'*<br>**INT 6:** *'GPs, locally they tend not to refer'*

5. Lack of resources / expertise

A few commissioners felt that service providers lacked expertise in conducting service evaluations.

- **INT 14:** *'there's difficulties there with the data that we need because we also find that the skill set of a lot of the people delivering the services doesn't always sit with evaluation'.*

Financial Pressures on Services

There are considerable financial pressures facing LAs at present and budget constraints are impacting on the provision of tier 2 weight management services for children in most LAs in different ways.

- **INT 17:** *'we're at a point now where we're going through council budget savings, the service has actually taken a 50% hit, which is huge'……………..' so how are we supposed to achieve this whole you know like city wide target on less money is going to be impossible'………'We've got smaller and smaller services and you keep telling me you're going to take some more money away from me so how are we supposed to achieve these things'*

Some LAs have found it challenging to provide a good service with reduced funding. Strategies taken to cope with the funding cuts have included setting lower targets as part of the key performance indicators (KPIs).

- **INT 1:** *'we've had to work together to reduce the KPIs anyway because they just wouldn't be met with that much of a dent in the finances'*

A few LAs are considering, or have already decided, to decommission their weight management service.

- **INT 2:** *'. So yeah things are really tight and at the regional network meeting people were talking that they may have to de-commission their weight management services'.*

For peer review only - http://bmjopen.bmj.com/site/about/guidelines.xhtml

LAs talked about the need to demonstrate 'good value for money' for a service to justify its funding.

- **INT 5:** *'I'm constantly looking at a cost benefit analysis and working out, okay how much is this costing per child, how much is it costing per family? What are the outcomes that we're getting? Is this really a programme that is cost effective?'*

A few LAs discussed the difficulties in allocating money to service evaluation when money for service provision itself was so limited.

Pressures on Service to Influence the Prevalence of Obesity

LAs often described the pressures they are under to reduce the prevalence of obesity within their borough through their tier 2 weight management programme. In some LAs, this seemed to be politically driven by councillors.

- **INT 2:** *'They're fixated about our actual prevalence rate'.....'the councillors yeah and sort of senior management. We've got like sort of corporate score card and they wanted to put obesity prevalence as part of that.'*

Reducing the prevalence of obesity was frequently seen as an unrealistic goal given the reach of the service often being so small, the funding allocated limited and the feeling that one service cannot be accountable for solely tackling such a complex problem with a programme length that is usually only 10-12 weeks.

- **INT 14;** *'In terms of tackling childhood obesity I'd say the child weight management programmes are family weight management programmes, they're only going to go so far. We know our population in LA14, we've probably got 500 families within each year group that would be affected by obesity even more that would be affected by overweight. If you times that by 18 years of childhood you've got quite a significant number of families up in the 10,000 maybe that are going to have these weight management issues. We're never going to be able to commission a service that would be able to work at a one to one level or a group level with 10,000 families, it's not going to be practical to do that. On the other side of things, we're looking at strategies that take a much more preventative approach.'*

To achieve the objective of reducing the prevalence of obesity, some LAs recognised that population-based approaches would be required.

- **INT 15:** *'the number of people we're getting to is actually quite small .... it's not going to change obesity levels locally, so we do need to look at more population-based approaches so that's something we will be doing... I suppose doing less programmes possibly in future becaus the numbers per programme aren't as high as we'd want'*

For peer review only - http://bmjopen.bmj.com/site/about/guidelines.xhtml

Need for a 'Whole Systems Approach'

Many of the LAs talked about a recent shift towards a 'whole systems approach' to tackling obesity [21] [22] [23] and the need to view weight management schemes alongside the 'bigger picture'.

- **INT 2:** *'we can run weight management schemes and I think they're really important, but it has to be part of the bigger picture because you know children's families only go to those sort of schemes like once a week. It's their whole environment that it's important to actually help them to making behaviour change and actually if we don't do both and try and change the obesogenic environment people aren't going to be successful in weight management and it's only going to be a short term, isn't it'*

Some felt that national strategies to try and change the obesogenic environment (e.g. active transport, sugar tax, change for life campaign) and perception of what constitutes a healthy weight were needed to influence the prevalence of obesity.

- **INT 3:** *'It's not going to be easy because it's more and more difficult to make healthy living the norm because it's just too easy to be unhealthy. It's going to take a major upheaval for it to get any better. I think the sugar taxes could help, I think we're going to see more and more of these. What I think we could do to improve it is more and more national campaigns, that's what I think'.*

Sharing and Use of Evaluation Data

Most LAs showed willingness to share data, however this tended to happen on an informal 'when requested' basis. Some LAs reported sharing data with other LAs more formally through obesity network meetings or emails, but this was on a regional rather than national level. Suggestions for future sharing mainly focused on developing online networks, forums or webinars which would enable data to be accessed both on a regional and national level.

- **INT 13:** *'I know in the sexual health areas they have like a forum or something, a website and they all sort of meet up and share best practice and they can ask questions online and things like that, so something like that for weight management would be good'*
- **INT 14:** *'I think there could be like a national monitoring ………………….It would be useful to be able to know exactly what data is needed and have methods for having that all collected in one place by one system and then to be able to pull reports from that system locally, regionally, sub-regionally, nationally and even if we could go down to a very local level even a ward level'.*

Some LAs felt that regional and national child obesity commissioner meetings would be useful. A few barriers mentioned to sharing data included time pressures, the commercially sensitive nature of some information and potential competition between LAs, though most did not feel that the latter was a significant issue.

For peer review only - http://bmjopen.bmj.com/site/about/guidelines.xhtml

Within LAs, evaluation or performance management data was mainly used to reshape and improve services and sometimes to promote the service and secure future funding.

Future Directions

1. Guidance needed on service specifications and contracts

Many LAs commented on the lack of consistency in service provider contracts, specifications and outcomes measured across different LAs. They felt that detailed practical guidance with sample service specifications and service provider contracts would be useful, including detailed guidance on what exactly the service should be aiming for in terms of weight loss and other objectives.

- **INT 19**: *I mean there's no like commissioning guidance on weight management programmes you know if that appears on my desk I'd be a very happy bunny because you know then it will tell me exactly what I need to look for, exactly what needs to be achieved. But we don't have a guidance that tells us that you know this is what you should expect from your provider.*
- **INT 17**: *I know trying to find some sort of consistency I think from a contracts point of view, it's been helpful that in other services, not children's weight management where we have had collaborative working around specifications and contracts and then obviously their local detail has been added to it.*

2. Cost Benefit Analyses Tool

In the current economic climate, a few LAs suggested that it would be helpful if researchers developed a cost-benefit analysis tool which they could use for their child weight management programmes to justify allocation of money to these programmes.

- **INT 18:** *'a cost analysis tool. So, in terms of if X loses 5% in terms of weight loss, what that saves NHS/CCG/whoever it may be long term, because we have these cost analysis tools for *another service within the LA*, we have GP cost per hour, things like that, but we don't have anything for weight management for young people, but a cost analysis tool would be great'.*

For peer review only

Discussion

Main Findings

Data from the online survey demonstrated that no LAs were aware of any peer-reviewed evidence supporting the effectiveness of their specific tier 2 weight management service at improving BMI centile. Service evaluations were not consistently conducted. There was little consistency in methods for reporting change in weight status. The mean cost of the service per child completing the intervention was £1,312 and the mean actual reach of the service (i.e. children completing the intervention) to overweight or obese children within a LA was only 1.2%.

The qualitative research revealed the complexities of 'in-house' contracts in some LAs. There were similarities between LAs in the length of the intervention programme commissioned, the timing of data collection points and the outcomes measured. There were inconsistencies in the tools used to measure these outcomes which complicates meaningful comparisons of data between LAs. Formal sharing of data between LAs was lacking. LAs identified many challenges facing their service in both provision, through lack of engagement and lack of resources, and in-service evaluation, through the questionable reliability of self-report data, lack of validated tools and difficulties in collecting long-term data.

Many LAs described the pressure on their service to reduce the prevalence of obesity but felt that a 'whole systems approach' was needed to tackle this problem rather than over-reliance on a single service. Some LAs felt more detailed guidance was needed on service specifications and contracts. Development of a cost-benefit analysis tool was also discussed by a few LAs.

Meaning of the Findings: Implications for policy makers and clinicians

There is currently no way of easily comparing BMI z-score or other outcome data between different tier 2 weight management programmes across multiple LAs in England. Although PHE have recently developed data entry forms, there is no mandatory system in place requiring LAs to submit this information so it can be collated onto a central database for analysis [24]. Where data are shared, this is usually done on an informal basis at a local level. This is surprising given that the online survey highlighted that no LAs knew of any peer-reviewed evidence supporting the effectiveness of their service at influencing weight status. In addition, there are very few UK-based research trials demonstrating a clinically significant reduction in BMI z-score in school aged children (defined as mean BMI SDS reduction of ≥ 0.25) [25-27]. A recent systematic review by Burchett et al reported only five of the thirty interventions included in the review reduced BMI z-score by ≥ 0.25 [26]. Of these five interventions, none was conducted in the UK and only 1 involved children of school-age.

Given the current economic climate and lack of evidence regarding long-term effectiveness of these interventions, it would seem wise to ensure that outcome data were being collected in a standardised format and that these data were compared and shared. This could help local and national agencies such as PHE to make evidence-based, cost-effective

For peer review only - http://bmjopen.bmj.com/site/about/guidelines.xhtml

commissioning decisions as the data in this paper suggests that these decisions are currently being conducted without good quality evidence of long-term benefit. However, even if this was achieved, many LAs have already alluded to the difficulties in collecting long-term data and so it is likely that there would be important gaps. It is also plausible that where long term data are collected, no long-term effectiveness is demonstrated. This is possible given that the NICE evidence review supporting the PH47 guideline reported no statistically significant mean difference in BMI z-score in the long-term for lifestyle weight management interventions for children [17].

Many LAs discussed the pressures on their service to reduce the prevalence of obesity. However, the actual mean reach of a service (i.e. children completing the intervention) to overweight or obese children within a LA was 1.2%. It is therefore unrealistic to expect these services to influence obesity prevalence rates. Population measures are needed to have population level effects and it is therefore unclear where Tier 2 services such as those evaluated fall within the overall obesity strategy as they are neither population focussed nor have a strong evidence base for clinically defined groups. Even if the service had the capacity to take a large proportion of the overweight or obese population, the programme still probably would not reach most of this population due to the difficulties in engagement discussed by LAs in the telephone interviews. Problems engaging families with services have been recognised in the literature [28]. Many LAs described the need for a 'whole-systems approach' to effectively tackle the problem of childhood obesity.

A whole systems approach recognises the need to address a complex multi-causal problem using multiple different approaches rather than through a single intervention alone [29 30 31]. On a LA level, this may involve influencing and linking multiple sectors such as planning, housing and transport, to effect population level changes [30 31]. Allender et al describe a community's understanding of the complex causality of obesity through a causal loop diagram [32] and they outline an obesity prevention trial aiming to use a whole systems community-led approach [33]. PHE have commissioned Leeds Beckett University to identify ways in which LAs might achieve a successful whole systems approach [31].

Weaknesses

The sample size for the online survey and telephone interviews were relatively small, but there was good geographical representation across England and saturation was felt to have been achieved in the telephone interviews. It is also not mandatory for LAs to commission a tier 2 weight management service, so some LAs may have felt this research was irrelevant. It is possible that in some LAs, details regarding the online survey did not reach the relevant person. A freedom of information (FOI) request was not submitted to obtain missing data and this is a limitation of the study.

Although a topic guide was used for the interviews, further discussions were guided by the participant. This had the strength of allowing inductive analyses to be conducted but the weakness that the opinions of every interview participant on each of the themes reported may not have been captured. It is also important to note that the current study focused on

For peer review only - http://bmjopen.bmj.com/site/about/guidelines.xhtml

LAs in England. This means that the generalisation of results to the rest of the UK and wider is unclear.

Finally, LAs did not provide answers in a comparable format for all questions on the online survey which limited statistical analyses to a relatively small number of LAs. This was likely in part due to variation in the type and format of data collected by each LA. A recent PHE study also recognised this problem, reporting that the average change in BMI centile post programme and at 12 months could not be determined due to the heterogeneity of respondents [34]. To gain a true oversight of the cost-effectiveness of lifestyle weight management programmes currently commissioned in the UK, there needs to be consistency in the outcomes measured and clear guidelines on what clinically significant outcomes these services should be aiming to achieve.

Strengths and Contextualisation

In 2015, PHE conducted a national mapping study of tier 2 and tier 3 weight management services [34]. The evidence-base for the commissioned service was determined by asking LAs if they followed NICE guidance or not, rather than asking whether their commissioned service had evidence supporting effectiveness in the peer-reviewed literature, as in this study. This is an important distinction as using guidelines to facilitate commissioning decisions is different to demonstrating the effectiveness of a commissioned service, especially given the limitations of the evidence supporting the NICE PH47 guidelines [35 36].

The PHE mapping study stated that the most frequently reported cost per participant of the service was ≥£401 though there is no further breakdown on figures above this range nor any standard deviations or mean costs provided. As a result, it is not possible to estimate the cost-effectiveness of these interventions. Furthermore, the survey asks for the 'average cost of the intervention per participant' but does not specify whether this should be per participant referred, per participant starting the intervention or per participant completing the intervention [37]. The strengths of our study are that we distinguish costs between these groups and report their means (with standard deviations).

In order to determine whether participants are followed up long-term, the PHE mapping study asked 'How long are the providers required to follow up the participants?' The study reported that 67% of services reported follow-up of participants for 12 months or more. However, being 'required' to follow-up doesn't mean that the data were collected for all these participants. Our qualitative data provides insight into the difficulties in collecting long-term data even when the specification to do so is present.

The qualitative aspect of the PHE study had some similarities with our research, reporting lack of evidence of long-term effectiveness, lack of validated tools, lack of clear guidance on specifications, lack of funding, lack of expertise and difficulties with recruitment.

Future Directions

In their present format, tier 2 weight management services for overweight and obese children are very unlikely to have any impact on the prevalence of childhood obesity and peer-reviewed evidence of any long-term benefits even for the small numbers of children

For peer review only - http://bmjopen.bmj.com/site/about/guidelines.xhtml

reached by these services, is weak. If these lifestyle weight management services are to be continued, clear thought needs to be given to the goals of the service and a more robust independent system needs to be developed to determine whether these goals are being met, whether the service is cost-effective and if it is the best use of limited resources in the current economic climate. Subsequently, if cost effectiveness is demonstrated, work needs to be undertaken to understand the variation in the provision of these services across England, such as through an 'Atlas of Variation [38],' and how LAs can be supported in the commissioning and delivery of these services, given that they are non-mandatory.

However, it is also important to consider whether preferential investment should be given to population level approaches or to developing strategies to deliver a whole systems approach by LAs rather than investing in a single small-scale, lifestyle weight management programme. Population measures such as the sugar tax, have been identified as having the potential to reduce the prevalence of obesity with the greatest benefit predicted for those under the age of 18 [39]. In Mexico, the tax on sugar sweetened beverages (SSBs) in 2013 was associated with fewer taxed beverages being bought and more untaxed beverages being bought [40]. A similar tax in California reduced SSB consumption in low-income neighbourhoods [41]. Yet, there is limited direct evidence of a link between a sugar tax and reduction in obesity prevalence aside from modelling studies. Other population level strategies include reduction of TV advertising of high fat and/or high sugar foods and drinks to children [42], nutritional labelling of foods, transport policies and multi-component mass media campaigns [43]. Nonetheless, Mckinsey et al suggest that public health campaigns have the least evidence for cost effectiveness [44].

Regardless of how funding is allocated to tackling obesity, there needs to be robust cost-effectiveness analyses and sharing of data nationally to help inform future commissioning decisions and ensure that scarce financial resources are being used in the most efficient and effective way across England.

Conclusion

Our results show that none of the participating LAs were aware of any peer-reviewed evidence supporting the effectiveness of their specific commissioned service. Despite this, there was no national formal sharing of data to enable oversight of the effectiveness of commissioned services across LAs in England to help inform future commissioning decisions. Challenges with long-term data collection, service engagement, funding and the pressure to reduce the prevalence of obesity were frequently mentioned. The need for a 'whole-systems approach' to tackle obesity effectively was discussed. In the future, obesity treatment or prevention programmes need to have robust systems in place to feedback programme outcomes and costs in a comparable and transparent format to enable national, independent oversight of the cost-effectiveness of different obesity strategies and direct future commissioning decisions.

Contributors and Guarantor: JHS, RJ, DS, RK and RM conceived and designed the study. Provisional analyses of the online survey data were conducted by RM and the telephone interview data by RM and AP. The results and analyses were reviewed and discussed by JHS,

For peer review only - http://bmjopen.bmj.com/site/about/guidelines.xhtml

RJ and DS. RM drafted the manuscript. All authors revised and approved the final manuscript. The corresponding author attests that all listed authors meet authorship criteria and that no others meeting the criteria have been omitted. RM is guarantor.

Funding Statement: RM is funded by the NIHR (NIHR ACF 2014-2017, NIHR In-Practice Fellowship 2018-2021). RK works in the Centre for the Development and Evaluation of Complex Interventions for Public Health Improvement (DECIPHer), a UKCRC Public Health Research Centre of Excellence: joint funding (MR/KO232331/1) from the British Heart Foundation, Cancer Research UK, Economic and Social Research Council, Medical Research Council, the Welsh Government and the Wellcome Trust, under the auspices of the UK Clinical Research Collaboration, is gratefully acknowledged. J.H-S. is supported by the National Institute of Health Research (NIHR) Biomedical Research Centre at the University Hospitals Bristol National Health Service (NHS) Foundation Trust and the University of Bristol, Bristol, UK. DS works in in CAPC PHS funded by HEE Severn.

Disclaimer: The views expressed in this publication are those of the authors and not necessarily those of the NHS, the National Institute for Health Research or the Department of Health and Social Care.

Copyright and Licence: The Corresponding Author has the right to grant on behalf of all authors and does grant on behalf of all authors, a worldwide licence (http://www.bmj.com/sites/default/files/BMJ%20Author%20Licence%20March%202013.doc) to the Publishers and its licensees in perpetuity, in all forms, formats and media (whether known now or created in the future), to i) publish, reproduce, distribute, display and store the Contribution, ii) translate the Contribution into other languages, create adaptations, reprints, include within collections and create summaries, extracts and/or, abstracts of the Contribution and convert or allow conversion into any format including without limitation audio, iii) create any other derivative work(s) based in whole or part on the on the Contribution, iv) to exploit all subsidiary rights to exploit all subsidiary rights that currently exist or as may exist in the future in the Contribution, v) the inclusion of electronic links from the Contribution to third party material where-ever it may be located; and, vi) licence any third party to do any or all of the above. All research articles will be made available on an open access basis (with authors being asked to pay an open access fee—see http://www.bmj.com/about-bmj/resources-authors/forms-policies-and-checklists/copyright-open-access-and-permission-reuse). The terms of such open access shall be governed by a Creative Commons licence—details as to which Creative Commons licence will apply to the research article are set out in our worldwide licence referred to above.

Competing Interest Statement*:* All authors have completed the ICMJE uniform disclosure form at www.icmje.org/coi_disclosure.pdf. During the conduct of this study, Dr. Mears and Professor Hamilton-Shield reports grants from National Institute of Healthcare Research, Professor Jago reports grants from NIHR and Dr. Kipping reports grants from DECIPHer, grant funding from a collection of charities and funding councils as outlined in the acknowledgments section of this paper. Professor Sharp and Ms Patel have nothing to disclose.

For peer review only - http://bmjopen.bmj.com/site/about/guidelines.xhtml

Patient and Public Participation: Patients/public were not involved in the design of this study or to interpret results.

Data Sharing Statement: Participants gave consent for collection of data which would then be anonymised, so it is not be possible to share individual LA data.

For peer review only

For peer review only - http://bmjopen.bmj.com/site/about/guidelines.xhtml

References

1. Collaboration NCDRF. Worldwide trends in body-mass index, underweight, overweight, and obesity from 1975 to 2016: a pooled analysis of 2416 population-based measurement studies in 128.9 million children, adolescents, and adults. *Lancet* 2017;390(10113):2627-42.
2. World Health Organisation. Tenfold increase in childhood and adolescent obesity in four decades: new study by Imperial College London and WHO http://www.who.int/mediacentre/news/releases/2017/increase-childhood-obesity/en/2017 (Accessed March 2019).
3. World Health Organisation. Global Health Observatory Data http://www.who.int/gho/ncd/risk_factors/overweight_obesity/overweight_adolescents/en/2016 (Accessed March 2019).
4. Singh AS, Mulder C, Twisk JW, et al. Tracking of childhood overweight into adulthood: a systematic review of the literature. *Obesity reviews : an official journal of the International Association for the Study of Obesity* 2008;9(5):474-88. doi: 10.1111/j.1467-789X.2008.00475.x [published Online First: 2008/03/12]
5. Finkelstein EA, Graham WC, Malhotra R. Lifetime direct medical costs of childhood obesity. *Pediatrics* 2014;133(5):854-62.
6. NICE. Weight management: lifestyle services for overweight or obese children and young people. PH47. https://www.nice.org.uk/guidance/ph47/history2013 (Accessed March 2019).
7. NHS England. Report of the working group into: Joined up clinical pathways for obesity https://www.england.nhs.uk/wp-content/uploads/2014/03/owg-join-clinc-path.pdf2014 (Accessed March 2019).
8. NHS. NHS structure explained https://www.nhs.uk/using-the-nhs/about-the-nhs/nhs-structure-explained/2016 (Accessed March 2019).
9. HM Government. Health and Social Care Act 2012: structures factsheet https://assets.publishing.service.gov.uk/government/uploads/system/uploads/attachment_data/file/138258/A3.-Factsheet-Overview-of-health-and-care-structures-240412.pdf2012 (Accessed March 2019).
10. NHS Digital. National Childhood Measurement Programme England 2014-2015 https://digital.nhs.uk/catalogue/PUB19109 (Accessed March 2019).
11. Public Health England. Weight management: guidance for commissioners and providers https://www.gov.uk/government/collections/weight-management-guidance-for-commissioners-and-providers2017 (Accessed March 2019).
12. Ford AL, Hunt LP, Cooper A, et al. What reduction in BMI SDS is required in obese adolescents to improve body composition and cardiometabolic health? *Archives of Disease in Childhood*;95(4):256-61.
13. Reinehr T, Lass N, Toschke C, et al. Which Amount of BMI-SDS Reduction Is Necessary to Improve Cardiovascular Risk Factors in Overweight Children? *Journal of Clinical Endocrinology & Metabolism*;101(8):3171-9.
14. Peirson L, Fitzpatrick-Lewis D, Morrison K, et al. Treatment of overweight and obesity in children and youth: a systematic review and meta-analysis. *CMAJ open*;3(1):E35-46.
15. Seburg EM, Olson-Bullis BA, Bredeson DM, et al. A Review of Primary Care-Based Childhood Obesity Prevention and Treatment Interventions. *Current Obesity Reports*;4(2):157-73.
16. Upton P, Taylor C, Erol R, et al. Family-based childhood obesity interventions in the UK: a systematic review of published studies. *Community Practitioner*;87(5):25-9.
17. F. Morgan AW, S. Whitehead, S. Brophy, H. Morgan, R. Turley, R. Kipping, J. White, W. Hollingworth Managing overweight and obesity among children and young people: lifestyle weight management services. Review 1: Effectiveness and cost effectiveness of lifestyle weight management services for children and young people. *NICE* 2013
18. The Kings Fund. Spending on public health https://www.kingsfund.org.uk/projects/nhs-in-a-nutshell/spending-public-health2018 (Accessed March 2019).

For peer review only - http://bmjopen.bmj.com/site/about/guidelines.xhtml

19. National Child Measurement Programme. NCMP- England, 2014-15: Tables https://digital.nhs.uk/data-and-information/publications/statistical/national-child-measurement-programme/2014-15-school-year2015 (Accessed March 2019).
20. Morse JM. "Data were saturated . . . ". *Qualitative Health Research* 2015;25(5):587-8.
21. Local Government Association. Whole Systems Approach to Obesity: Co-creating a world class solution to a world wide challenge https://www.local.gov.uk/sites/default/files/documents/P11%20Using%20whole%20systems%20approaches%20to%20tackle%20obesity.pdf (Accessed March 2019).
22. Local Government Association. Making obesity everybody's business: A whole systems approach to obesity https://www.local.gov.uk/making-obesity-everybodys-business-whole-systems-approach-obesity2017 (Accessed March 2019).
23. House of Commons Health Committee. Eight Report of Session 2017-19. Childhood Obesity: Time for Action https://publications.parliament.uk/pa/cm201719/cmselect/cmhealth/882/882.pdf2018 (Accessed March 2019)
24. Public Health England. Child weight management services: collect and record data. Capturing data: a tool to collect and record child weight management services data. https://www.gov.uk/government/publications/child-weight-management-services-collect-and-record-data2017 (Accessed March 2019).
25. Al-Khudairy L, Loveman E, Colquitt JL, et al. Diet, physical activity and behavioural interventions for the treatment of overweight or obese adolescents aged 12 to 17 years. *Cochrane Database of Systematic Reviews*;6:CD012691.
26. Burchett HED, Sutcliffe K, Melendez-Torres GJ, et al. Lifestyle weight management programmes for children: A systematic review using Qualitative Comparative Analysis to identify critical pathways to effectiveness. *Preventive Medicine*;106:1-12.
27. Mead E, Brown T, Rees K, et al. Diet, physical activity and behavioural interventions for the treatment of overweight or obese children from the age of 6 to 11 years. *Cochrane Database of Systematic Reviews*;6:CD012651.
28. Kelleher E, Davoren MP, Harrington JM, et al. Barriers and facilitators to initial and continued attendance at community-based lifestyle programmes among families of overweight and obese children: a systematic review. *Obesity Reviews*;18(2):183-94.
29. Lee BY, Bartsch SM, Mui Y, et al. A systems approach to obesity. *Nutrition Reviews*;75(suppl 1):94-106.
30. Rutter H, Savona N, Glonti K, et al. The need for a complex systems model of evidence for public health. *Lancet*;390(10112):2602-04.
31. Leeds Beckett University. Whole Systems Approach to Tackle Obesity http://www.leedsbeckett.ac.uk/wholesystemsobesity/: Leeds Beckett University (Accessed March 2019).
32. Allender S, Owen B, Kuhlberg J, et al. A Community Based Systems Diagram of Obesity Causes. *PLoS ONE [Electronic Resource]*;10(7):e0129683.
33. Allender S, Millar L, Hovmand P, et al. Whole of Systems Trial of Prevention Strategies for Childhood Obesity: WHO STOPS Childhood Obesity. *International Journal of Environmental Research & Public Health [Electronic Resource]* 2016;13(11):16.
34. Public Health England. National Mapping of Weight Management Services, 2015 https://assets.publishing.service.gov.uk/government/uploads/system/uploads/attachment_data/file/484115/Final_Weight_Management_Mapping_Report.pdf (Accessed March 2019).
35. Morgan F WA, Whitehead S, Brophy S, Morgan HE, Turley RL, Kipping R, White J, Hollingworth W. . Managing overweight and obesity among children and young people: Review 1 - Effectiveness and cost effectiveness of lifestyle weight management services for children and young people. In: NICE, ed., 2013.

For peer review only - http://bmjopen.bmj.com/site/about/guidelines.xhtml

36. Turley RL WA, Halstead E, Morgan HE, Morgan F, Noyes J, Brophy S, Kipping R and Whitehead S. . Managing overweight and obesity among children and young people: lifestyle weight management services. Review 2: The barriers and facilitators to implementing lifestyle weight management programmes for children and young people. In: NICE, ed., 2012.
37. Public Health England. National mapping of weight management services: e-survey and face to-face approach, 2015 https://assets.publishing.service.gov.uk/government/uploads/system/uploads/attachment_data/file/768890/National_mapping_of_weight_management_services_Annex_2.pdf (Accessed March 2019)
38. Public Health England. Atlas of Variation https://fingertips.phe.org.uk/profile/atlas-of-variation (Accessed March 2019).
39. Briggs ADM, Mytton OT, Kehlbacher A, et al. Health impact assessment of the UK soft drinks industry levy: a comparative risk assessment modelling study. *The lancet Public Health*;2(1):e15-e22.
40. Colchero MA, Popkin BM, Rivera JA, et al. Beverage purchases from stores in Mexico under the excise tax on sugar sweetened beverages: observational study. *BMJ*;352:h6704.
41. Falbe J, Thompson HR, Becker CM, et al. Impact of the Berkeley Excise Tax on Sugar-Sweetened Beverage Consumption. *American Journal of Public Health*;106(10):1865-71.
42. Haby MM, Vos T, Carter R, et al. A new approach to assessing the health benefit from obesity interventions in children and adolescents: the assessing cost-effectiveness in obesity project. *International Journal of Obesity*;30(10):1463-75.
43. World Health Organisation. Population-based approaches to childhood obesity prevention https://www.who.int/dietphysicalactivity/childhood/WHO_new_childhoodobesity_PREVENTION_27nov_HR_PRINT_OK.pdf2012 (Accessed March 2019).
44. Dobbs R SC, Thompson F, Manyika J, Woetzel J, Child P, Mckenna S and Spatharou A. Overcoming obesity: an initial economic assessment. https://www.mckinsey.com/~/media/mckinsey/business%20functions/economic%20studies%20temp/our%20insights/how%20the%20world%20could%20better%20fight%20obesity/mgi_overcoming_obesity_full_report.ashx: McKinsey Global Institute; 2014 (Accessed March 2019).

For peer review only - http://bmjopen.bmj.com/site/about/guidelines.xhtml

Supplementary File 1 – Online Survey Questions:

National Survey of LA Commissioned Weight Management Services for Overweight / Obese Children in 2014/15

	**Informed Consent for Online Survey**
	In order to take part in this online survey, we need to obtain your informed consent. Please read the following five statements carefully.<br>1. I confirm that I have read and understood the participant information.<br>2. I am willing to take part in the survey<br>3. I understand that my name will be kept anonymous however due to the nature of the study, it is not possible to anonymise the local authority name<br>4. I understand that information collected (name of survey participant anonymised but name of local authority not anonymised) will be stored for 10 years in data sets within a secure facility in accordance with the Data Protection Act 1998 and this data may be used in publications or presentations to relevant audiences or shared with other researchers.<br>5. I understand that I can withdraw from the study at any point prior to March 21st 2016 by emailing Dr Ruth Mears on rm14101@bristol.ac.uk
**Question 1**	Please confirm that you have read, understood and agree to the above five statements
**Response 1**	
	**Participant & Local Authority Details**
**Question 2**	What local authority do you work in?<br>*Please note that if you work for multiple local authorities, you will need to fill out a new survey for each local authority that you work for.*
**Response 2**	
	**Tier 2 Weight Management Services for Overweight/Obese Children in your Local Authority**
**Question 3**	Please name a Tier 2 weight management service commissioned by your local authority for overweight / obese children aged 4-16 years during the financial year April 2014-March 2015?
**Response 3**	
**The following questions relate to the Tier 2 weight management service you have named above.**	
**Question 4**	What evidence are you aware of regarding the effectiveness of the service commissioned between April 2014 – March 2015 at reducing BMI centile / BMI % / BMI z-score or BMI? Please choose (highlight) from the below list;
**Response 4**	– *Data published in a peer reviewed journal – independently collected (i.e. data collected by a person who is NOT an employee of the weight management service provider)*

For peer review only

	– *Data published in a peer reviewed journal – internally collected (i.e. data collected by a person who IS an employee of the weight management service provider)*<br>– *Published in an alternative source – independently collected (i.e. data collected by a person who is NOT an employee of the weight management service provider)*<br>– *Published in an alternative source – internally collected (i.e. data collected by a person who IS an employee of the weight management service provider)*<br>– *Unpublished data*<br>– *Other*<br>– *No evidence*
**Question 5**	Please specify where the evidence can be found regarding the effectiveness of the services commissioned during the year April 2014-March 2015 at reducing BMI centile / BMI % / BMI z-score or BMI?<br>*(e.g. publication details / website address etc. If the data is unpublished, please email details to rm14101@bristol.ac.uk)*
**Response 5**	
**Question 6**	Since the service was commissioned (i.e. contract start date), had it been evaluated within your local authority?
**Response 6**	
**Question 7**	As part of the service evaluation, was change in weight status measured (e.g. change in BMI, BMI%, BMI centile or BMI z-score?)
**Response 7**	
**Question 8**	If change in weight status was measured, what were the results?<br>*Please write the time frame in which this change occurred e.g. Reduction of BMI centile by 0.9% (SD) over 1 year (2014/15). If this is available for different age groups, please indicate the results by age group.*
**Response 8**	
**Question 9**	What was the total cost of the service for the local authority between April 2014 to March 2015?<br>*If data cannot be provided please specify the time period and costs in the format you have available e.g. cost per child per course in August 2014.*
**Response 9**	
**Question 10**	What was the maximum number of participants that could have been accommodated by the commissioned service between April 2014 to March 2015?<br>*Where possible, please provide data on maximum commissioned capacity for a one year time frame from 2014 to 2015. If this data cannot be provided, please specify the time period and maximum capacity of the service in the format you have available e.g. maximum capacity of 60 children per course in 2014, total of 10 courses in 2014.*

**Response 10**	
**Question 11**	How many children were referred to the service between April 2014 to March 2015?<br>*If this data cannot be provided please specify the time period and referral data in the format you have available*
**Response 11**	
**Question 12**	How many children completed the intervention between April 2014 to March 2015?<br>*If data cannot be provided please specify the time period and number completing the intervention in the format you have available*
**Response 12**	
	**Thank you for taking the time to complete this survey. Please consider taking part in the second phase of our research.**
**Question 13**	Thank you for taking the time to complete this survey.<br><br>Would you like to receive a summary of the results and analysis by email?<br><br>If you answered yes to the above question, please provide us with your email address
**Response 13**	
**Question 14**	If you answered yes to the above question, please provide us with your email address
**Response 14**	
	**Telephone Interview**

The second phase of our research will involve a telephone interview exploring commissioners' views and experiences in the evaluation of weight management services for overweight and obese children. There is little qualitative evidence available regarding service evaluation data collected by commissioners and this research aims to fill the gap in the literature. We will explore the views of commissioners on the role and value of service evaluation data, the barriers and facilitators to collecting and processing this data and finally how to ensure evaluation data is useful. Performance management of services will also be explored.

If you are interested in participating in the interview, please can you provide your name and contact details (email and/or telephone number) below. We will then email you a participant information sheet providing further details about what the interview involves. After reading the information sheet, if you decide you would like to take part, you will need to fill out the consent form and send it to rm14101@bristol.ac.uk. Dr Ruth Mears will then contact you to arrange a convenient time for you to conduct the telephone interview

……

……

……

For peer review only

Supplementary File 2

Interview Guide: Commissioning and evaluation of lifestyle weight management programmes in England

**Icebreaker**	**Prompts**
What is your role in the local authority?	*How long have you been in this role?*
How are you involved in the commissioning of weight management services for obese and overweight children?	*What is your background? Public Health?*
**Part One: Role and value of evaluations**	
What do you understand by the term 'service evaluation'?	*Why conduct a service evaluation? Role for commissioners? Role for participants? Role for service providers?*
What do you understand by the term 'performance management'?	*Why collect performance management data?*
How do you think service evaluation and performance management differ?	
How important do you think service evaluation is?	*Importance to commissioners / LA / personal opinions? Why? What informs the decision to undertake a service evaluation (e.g. pilot, new service, lack of evidence, review, retenderin)?*
How useful do you think service evaluation is?	*Why? What parts are useful? What parts are not useful? Do your personal views differ from what you feel are the views of the local authority?*
What outcomes are currently viewed as a measure of success by your local authority for a childhood weight management programme?	*Do you agree that this outcome should be viewed as a measure of success? What are your personal views? Which outcomes do you think are the most important? Why?*
What outcome data from a service evaluation is viewed as essential by your local authority? *(or performance management data if service evaluations have not been conducted)*	*Why? Do you agree?*
What information from a service evaluation does your local authority least value? *(or performance management data if service evaluations have not been conducted)*	*Why? Do you agree?*

For peer review only

Supplementary File 2

**Part Two: LA Specific Questions**	
Reflecting on a weight management service you have been involved in, can you tell me how the service was evaluated?<br>*If the service has not been evaluated, please can you tell me about performance management of the service.*	*Who collected the evaluation data (external evaluator or internal person)? At what time points was the data collected? What data was collected? Was a specific evaluation data collection tool used?*
What went well in collecting evaluation data *(or performance management data if no service evaluation has been conducted)*?	*What barriers were there? What are the weaknesses in your data? What are the strengths of your data? Were service providers happy to co-operate with the evaluation process? Were there any difficulties in collecting data from service users? How could service evaluation data be improved?*
What did not go well in collecting evaluation data *(or performance management data if no service evaluation has been conducted)*?	
Do you think the data collected was useful?	***Yes:*** *In what way was it useful? How could it have been more useful? How were the data used?*<br>***No:*** *Why do you think it was not useful? Is there are anything which would have made it more useful?*
How were the service evaluation data *(or performance management data if no service evaluation has been conducted)* used by your local authority?	*By commissioners? By service providers? To improve services? To inform future commissioning decisions? Is this reflective of how other service evaluation data has been used*
How do you think the information collected from a service evaluation *(or performance management data if no service evaluation has been conducted)* should be used for maximum benefit?	*By commissioners? By service providers?*
**Part Three: Improving the process of service evaluation and use of outcome data**	
What resources / tools / information / guidance are currently available to help commissioners conduct service evaluations?	*Are they useful? What are the downsides of them? What would you find useful to have which is not currently available?*
Do you have access to online journals - OVID / Medline databases etc?	*Would you feel comfortable reviewing evidence from these databases?*
Who is responsible for collecting the service evaluation *(or performance management)* outcome data in your local authority?	*If it is the service providers, do you think they should be? Why? Why not?*
What are your opinions on the sharing of evaluation *(or performance data)* between local authorities and other organisations?	*Do you have any reservations? Do you think it would be beneficial or*

For peer review only

Supplementary File 2

	detrimental? Use in future commissioning decisions?
Do you share evaluation *(or performance data)* with other local authorities or organisations?	***Yes:*** *How do you do this? Is the data actively shared? Is it useful? Is the data shared of good quality? Does it play a role in future commissioning decisions?*<br>***No:*** *Why not? Do you think data should be shared? What are the barriers to sharing data?*
Do you think service contracts should be based on performance?	*E.g. must attain x change in BMI percentile or the contract will be terminated. What are the benefits of linking service contracts to performance? What are the downsides of this?*
In your Local Authority, are service evaluations used to identify underperforming services?	***Yes:*** *Do you think they should be used in this way? If a weight management service within your local authority is underperforming, are there any procedures which you would follow?*<br>***No:*** *Do you think they should be used in this way?*
How do you think the process of service evaluation *(or performance data)* and use of outcome data could be optimised to ensure maximum benefit for commissioners?	*Maintaining standards? Future decisions? Sharing?*
Are you aware of any recent national (NOO or NICE) guidelines regarding the evaluation of lifestyle weight management programmes?	*What do you know about them? At what time points do these guidelines recommend collecting outcome data? Does your local authority currently collect data at this timepoint? Is your local authority planning to implement these guidelines?*
What information do you think is needed to assist commissioners in providing successful weight management programmes?	*(Prompts; research? evidence? guidelines?)*
**Closing**	
That's all the questions I have for you today. Do you have any other comments you wish to make about service evaluations?	
Do you have any questions for me?	
Thank you very much for your time and attention. We appreciate you sharing your thoughts and opinions with us.	

For peer review only

COREQ (COnsolidated criteria for REporting Qualitative research) Checklist

A checklist of items that should be included in reports of qualitative research. You must report the page number in your manuscript where you consider each of the items listed in this checklist. If you have not included this information, either revise your manuscript accordingly before submitting or note N/A.

Topic	Item No.	Guide Questions/Description	Reported on Page No.
**Domain 1: Research team and reflexivity**			
Personal characteristics			
Interviewer/facilitator	1	Which author/s conducted the interview or focus group?	Pg 3
Credentials	2	What were the researcher's credentials? E.g. PhD, MD	MBBCh, BSc
Occupation	3	What was their occupation at the time of the study?	GP Registrar, NIHR ACF
Gender	4	Was the researcher male or female?	Female
Experience and training	5	What experience or training did the researcher have?	MBBCh, BSc
Relationship with participants			
Relationship established	6	Was a relationship established prior to study commencement?	No
Participant knowledge of the interviewer	7	What did the participants know about the researcher? e.g. personal goals, reasons for doing the research	RM working part-time in general practice and part-time in research
Interviewer characteristics	8	What characteristics were reported about the inter viewer/facilitator? e.g. Bias, assumptions, reasons and interests in the research topic	As above
**Domain 2: Study design**			
Theoretical framework			
Methodological orientation and Theory	9	What methodological orientation was stated to underpin the study? e.g. grounded theory, discourse analysis, ethnography, phenomenology, content analysis	Pg 4, Inductive thematic analysis
Participant selection			
Sampling	10	How were participants selected? e.g. purposive, convenience, consecutive, snowball	Pg 3
Method of approach	11	How were participants approached? e.g. face-to-face, telephone, mail, email	Pg 5
Sample size	12	How many participants were in the study?	Pg 6
Non-participation	13	How many people refused to participate or dropped out? Reasons?	From the online survey, 31 expressed an interest to participate in the telephone interviews.

For peer review only

Setting			
Setting of data collection	14	Where was the data collected? e.g. home, clinic, workplace	Workplace
Presence of nonparticipants	15	Was anyone else present besides the participants and researchers?	Nil with RM. Interview participants may be sharing an office.
Description of sample	16	What are the important characteristics of the sample? e.g. demographic data, date	Pg 6
Data collection			
Interview guide	17	Were questions, prompts, guides provided by the authors? Was it pilot tested?	Pg 3
Repeat interviews	18	Were repeat inter views carried out? If yes, how many?	No
Audio/visual recording	19	Did the research use audio or visual recording to collect the data?	Pg 4
Field notes	20	Were field notes made during and/or after the inter view or focus group?	No
Duration	21	What was the duration of the inter views or focus group?	Pg 6
Data saturation	22	Was data saturation discussed?	Pg 4
Transcripts returned	23	Were transcripts returned to participants for comment and/or correction?	No
**Topic**	**Item No.**	**Guide Questions/Description**	**Reported on Page No.**
**Domain 3: analysis and findings**			
Data analysis			
Number of data coders	24	How many data coders coded the data?	Pg 4
Description of the coding tree	25	Did authors provide a description of the coding tree?	No
Derivation of themes	26	Were themes identified in advance or derived from the data?	Pg 4
Software	27	What software, if applicable, was used to manage the data?	Pg 4
Participant checking	28	Did participants provide feedback on the findings?	Not yet. Summary will be sent to participants in due course.
Reporting			
Quotations presented	29	Were participant quotations presented to illustrate the themes/findings? Was each quotation identified? e.g. participant number	Pg 6-13
Data and findings consistent	30	Was there consistency between the data presented and the findings?	Pg 14-17
Clarity of major themes	31	Were major themes clearly presented in the findings?	Pg 6-13
Clarity of minor themes	32	Is there a description of diverse cases or discussion of minor themes?	Pg 6-13

Developed from: Tong A, Sainsbury P, Craig J. Consolidated criteria for reporting qualitative research (COREQ): a 32-item checklist for interviews and focus groups. *International Journal for Quality in Health Care*. 2007. Volume 19, Number 6: pp. 349 – 357

For peer review only

Once you have completed this checklist, please save a copy and upload it as part of your submission. DO NOT include this checklist as part of the main manuscript document. It must be uploaded as a separate file.

For peer review only

BMJ Open

A mixed methodology study exploring how lifestyle weight management programmes for children are commissioned and evaluated in England.

Journal:	*BMJ Open*
Manuscript ID	bmjopen-2018-025423.R2
Article Type:	Research
Date Submitted by the Author:	15-Sep-2019
Complete List of Authors:	Mears, Ruth; Centre for Academic Primary Care, Bristol Medical School, University of Bristol,; Centre for Exercise Nutrition and Health Sciences, School for Policy Studies, University of Bristol,<br>Jago, Russ; Centre for Exercise Nutrition and Health Sciences, School for Policy Studies, University of Bristol,<br>Sharp, Deborah; Centre for Academic Primary Care, Bristol Medical School, University of Bristol,<br>Patel, Anamica; Centre for Exercise Nutrition and Health Sciences, School for Policy Studies, University of Bristol<br>Kipping, Ruth; Department of Population Health Sciences, Bristol Medical School, University of Bristol,; Centre for the Development and Evaluation of Complex Interventions for Public Health Improvement (DECIPHer),<br>Shield, Julian; NIHR Bristol Biomedical Research Centre, Nutrition Theme, University of Bristol,; Faculty of Health Sciences, University of Bristol, BS2 8DZ, UK
**Primary Subject Heading**:	Public health
Secondary Subject Heading:	Evidence based practice, General practice / Family practice, Health services research, Nutrition and metabolism, Paediatrics
Keywords:	obesity, lifestyle weight management services, children, commissioning, evaluation

SCHOLARONE™
Manuscripts

A mixed methodology study exploring how lifestyle weight management programmes for children are commissioned and evaluated in England

Mears R [1,2], Jago R [2], Sharp D [1], Patel A[2], Kipping R [3,4], Shield JPH [5,6]

Centre for Academic Primary Care, Bristol Medical School, University of Bristol, BS8 2PS, UK [1]. Centre for Exercise Nutrition and Health Sciences, School for Policy Studies, University of Bristol, BS8 1TZ, UK [2]. Department of Population Health Sciences, Bristol Medical School, University of Bristol, BS8 2PS, UK [3]. Centre for the Development and Evaluation of Complex Interventions for Public Health Improvement (DECIPHer), Bristol, BS8 2PS, UK [4]. NIHR Bristol Biomedical Research Centre, Nutrition Theme, University of Bristol, BS1 2NT, UK [5]. Faculty of Health Sciences, University of Bristol, BS2 8DZ, UK [6].

Corresponding Author: Dr Ruth Mears, rm14101@bristol.ac.uk

Author Positions: Dr Ruth Mears – NIHR In-Practice Fellowship in Primary Care. Professor Russell Jago - Professor of Paediatric Physical Activity and Public Health. Professor Debbie Sharp - Professor of Primary Health Care. Ms. Anamica Sharma - MSc Student. Ms. Ruth Kipping - Consultant Senior Lecturer in Public Health. Professor Hamilton-Shield - Professor in Diabetes and Metabolic Endocrinology.

For peer review only

For peer review only - http://bmjopen.bmj.com/site/about/guidelines.xhtml

Abstract

Objectives: To assess how lifestyle weight management programmes for children aged 4-16 years in England are commissioned and evaluated at the local level.

Design: This was a mixed methods study comprising an online survey and semi-structured telephone interviews.

Setting: An online survey was sent to all Local Authorities (LAs) in England regarding lifestyle weight management services commissioned for children aged 4-16 years. Online survey data were collected between February and May 2016 and based on services commissioned between April 2014 and March 2015. Semi-structured telephone interviews with LA staff across England were conducted between April and June 2016.

Participants: Commissioners or service providers working within the Public Health Department of LAs.

Main outcome measures: The online survey collected information on the evidence-base, costs, reach, service usage and evaluation of child lifestyle weight management services. The telephone interviews explored the nature of child weight management contracts commissioned by LAs, the type of outcome data collected and whether these data were shared with other LAs or organisations, the challenges faced by these services and the perceived 'markers of success' for a programme.

Results: The online survey showed that none of the participating LAs were aware of any peer-reviewed evidence supporting the effectiveness of their specific commissioned service. Despite this, the telephone interviews revealed that there was no national formal sharing of data to enable oversight of the effectiveness of commissioned services across LAs in England to help inform future commissioning decisions. Challenges with long-term data collection, service engagement, funding and the pressure to reduce the prevalence of obesity were frequently mentioned.

Conclusions: Robust independent, cost-effectiveness analyses of obesity strategies are needed to determine the appropriate allocation of funding to lifestyle weight management treatment services, population-level preventative approaches or development of whole-system approaches by an LA.

Strengths and limitations of this study

- There has been no previous independent, peer-reviewed research study assessing how lifestyle weight management programmes in childhood are being commissioned and evaluated across Local Authorities (LAs) in England.
- The response rate for the online survey was lower than desired however there was good geographical representation across England.
- The current study focused on LAs in England so generalisation of results to the rest of the UK and wider is unclear.
- The change in weight status and cost data provided by LAs precluded meaningful statistical analyses so it is impossible to comment on the cost-effectiveness of, or between, commissioned services.

For peer review only - http://bmjopen.bmj.com/site/about/guidelines.xhtml

For peer review only

- There were no freedom of information requests submitted to LAs who did not complete the online survey and it is possible further data could have been obtained through this route.

Introduction

In the past four decades, there has been a tenfold increase in the number of obese children and adolescents worldwide [1 2]. In the United Kingdom, 31.1% of children and adolescents were classified as overweight or obese in 2016 [3]. These children and adolescents are more likely to become overweight or obese adults and suffer health related consequences [4]. This presents a major public health challenge [5]. In the UK, weight management strategies are classified into tier 1 (those that focus on preventing obesity), tier 2 (lifestyle weight management services), tier 3 (specialist obesity services) and tier 4 services (pharmacological or surgical treatments for obesity) [6]. Tier 1 and 2 services are commissioned by public health departments working within Local Authorities (LAs). Clinical commissioning groups (CCG's) are responsible for commissioning Tier 3 services since 2014 and Tier 4 services since 2017 [7]. CCG's are responsible for the planning and commissioning of health care services for their local area and are assured by NHS England [8]. In 2013, Public Health England (PHE) was formed as a separate entity to NHS England as public health care transitioned from the NHS to LAs under the Health and Social Care Act 2012 [9].

This paper focuses on tier 2 weight management services commissioned by LAs across England for school-aged children (aged 4-16 years). There are 152 LAs in England [10] and each LA may choose to commission services from a different tier 2 service provider. Although there is guidance from the National Institute of Clinical Excellence (NICE) and PHE regarding what these services should comprise [6 11], the specific weight management programmes have rarely been independently evaluated and published. Furthermore, there are very few UK-based, randomised trials in the peer-reviewed literature demonstrating a clinically significant reduction in BMI z-score (defined as minimum BMI SDS reduction of ≥ 0.25)[12 13] through lifestyle weight management programmes alone for school aged-children [14 15 16]. Even the evidence reviews supporting the NICE public health guidance (PH47), only reported a post-intervention pooled reduction in BMI z-score of -0.17 (95% CI = -0.3 to -0.04, p=0.01) which was attenuated when long-term data (≥ 6 months) were used (Standardised Mean Difference (SMD) = -0.07; CI 95% = −0.15 to 0.02, p = 0.12) [17].

LAs usually monitor their tier 2 weight management services through 'Performance Management' meetings, although they may also conduct service evaluations. NICE recommends that monitoring focuses on sustaining long term changes [6], despite their evidence reviews showing little efficacy for these interventions in the long-term [17]. Given the poor evidence-base for tier 2 weight management services, it is important to understand more about the nature of the contracts commissioned by LAs, the monitoring of outcomes and the challenges facing these services. In addition, given the current financial climate in public health, with spending estimated by the King's Fund to be 8% lower four

For peer review only

years after public health moved from the NHS to LAs [18], it is important to explore whether these services are a good use of limited resources.

This mixed methods study uses quantitative methods (an online survey) to determine the evidence-base underpinning the local service provided, costs, reach, service usage and evaluation of tier 2 weight management programmes commissioned by LAs across England for children aged 4-16 years. Qualitative methods (semi-structured telephone interviews) explore the nature of childhood tier 2 weight management contracts commissioned by LAs, the type of outcome data collected and whether these data are shared, the challenges faced by these services and the perceived 'markers of success' for a programme. Finally, the data collected from both the online survey and telephone interviews examine whether lifestyle weight management programmes are a good use of limited resources.

For peer review only

For peer review only - http://bmjopen.bmj.com/site/about/guidelines.xhtml

Methods

Participants and Recruitment

A list of all 152 LAs in England was derived from 2014/2015 National Child Measurement Programme (NCMP) datasets [19]. The Director for Public Health for each LA was contacted by email and asked to identify the relevant person within their LA responsible for the commissioning of childhood tier 2 weight management services. An email was sent to this person asking if they would be willing to participate in an online survey exploring tier 2 weight management services for school-aged children commissioned between April 2014 and March 2015. If no response to the email was received, a further email was sent.

The final page of the online survey provided information about the second phase of the study (telephone interviews) and invited those interested in taking part to leave their contact details. In addition, some of those LAs who declined to participate in the online survey, were also invited by email to take part in the telephone interviews.

Patient and Public Involvement

Patients and public were not involved in the design of this study or to interpret results.

Design of Online Survey and Telephone Interview Guide

The online survey (Supplementary File 1) and interview guide (Supplementary File 2) were developed by RM, RJ, DS, JHS and RK. Development of the survey and interview guide were informed by the collective experiences of these clinicians and researchers in the field of childhood weight management and through addressing gaps in the current literature.

Ethical Approval

Ethical approval was granted by the School for Policy Studies Research Ethics Committee at the University of Bristol. Informed consent was obtained in written format for the online survey and in written or verbal format for the telephone interviews.

Online Survey

The online survey comprised 10 questions relating to tier 2 weight management services commissioned by the LA for overweight or obese children aged 4-16 years in March 2014 to April 2015. The survey collected data on the evidence-base supporting the commissioned intervention, the cost of the service, the maximum number of participants the service could have accommodated, the number of children referred, the number of children completing the intervention, whether a service evaluation had been conducted and the changes in weight status measured through service evaluation. Data were collected between February and May 2016 and analysed in Microsoft Excel.

Telephone Interviews

Semi-structured telephone interviews were conducted by RM between April and June 2016. The interview guide had a common framework but was adapted during the interview as guided by the participants' responses.

For peer review only - http://bmjopen.bmj.com/site/about/guidelines.xhtml

The interviews required participants to reflect on their experiences of tier 2 weight management services for school-aged children within their LA but was not confined to experiences within the time-period specified in the online survey of March 2014-April 2015. This enabled a broader representation of experiences from interview participants. The interviews explored the nature of the contracts commissioned by LAs and the monitoring of these services through performance management and service evaluation. Specifically, the interviews explored whether outcome data were collected, whether these data were shared, the challenges identified through monitoring processes and the perceived 'markers of success' for the service.

Interviews were audio-recorded then transcribed verbatim by Bristol Transcription Service. All interview transcripts were anonymised by AP and uploaded to N-vivo 10.0 for inductive thematic analysis.

Data were organised into codes and themes and constantly revised and reviewed by two researchers working independently (RM and AP). Once coding was complete, both researchers discussed differences and links within and across themes before agreeing on the final themes. Themes were inductively and deductively elicited based on the interview guide and the information that emerged during the interviews. Data saturation was deemed to have been met when no new information emerged from the interviews which resulted in a sample of 20 participants [20].

Transparency statement

The online survey was conducted as originally planned. The telephone interviews initially aimed to explore service evaluation and performance management of tier 2 weight management services for children from a commissioner's perspective and experiences. As it emerged that some LAs run in-house contracts, participants were included who were within a LA but also service providers. The data which subsequently emerged focused the analysis on determining whether lifestyle weight management programmes were a good use of limited resources.

For peer review only

Results

Quantitative Data from Online Survey

Survey Respondents

Contact details for 103 LA 'obesity leads' were obtained through Directors of Public Health and via suggestions from PHE. Of these, 40 completed the survey, 24 declined to complete the survey and provided a reason (nil commissioned n=14, service decommissioned n=4, insufficient resources to complete the survey n=3, declined for other reasons n=3) and 39 did not complete the survey and did not provide a reason. Of the remaining 49 LAs, it is possible that the DPH forwarded our email onto the relevant contact but did not copy us in or that some of these LAs simply did not commission a tier 2 weight management service for children.

Geographical Location of Survey Respondents

The geographical location of the forty LAs who completed the online survey were; North West (n=10), North East (n=2), Yorkshire and the Humber (n=4), West Midlands (n=3), East Midlands (n=1), East of England (n=3), London (n=7), South West (n=7) and South East (n=3). The population of children aged 4-16 years within each of these forty LAs ranged from 16,000 to 186,000 (Mid-2014 Population Data from Office of National Statistics).

Evidence-base of Tier 2 Weight Management Service Commissioned

No LAs were aware of evidence published in peer-reviewed journals demonstrating that their service was effective at improving BMI centile (or other weight related measure). Service evaluations were conducted in 55% of LAs, of which 18% did not measure change in weight status as part of their service evaluation. Due to heterogeneity in the way in which outcome data for change in weight status were reported by LAs (e.g. proportion who reduced or maintained their BMI z-score, number who 'lost weight', % of children who reduced their BMI z-score by 3%, only 6 or 12 month data), it was not possible to make any meaningful interpretations or comparisons of these data.

Costs and Reach of the Service

Table 1 summarises the costs of the service. Some LAs were only able to provide estimates. Table 2 summarises the reach of services within an LA.

Table 1: Costs of the Service

	Mean cost (SD, n = number of LAs providing data)
Cost of the service per year to LA	£130,742 (SD £122,869, n=27)
Cost of the service per year per 10,000 children aged 4-16 years (of any weight) in LA	£29,397 (SD £30,003, n=27)

For peer review only - http://bmjopen.bmj.com/site/about/guidelines.xhtml

Cost of the service per overweight or obese child attending if maximum capacity of the service was reached	£558 (SD £408, n=18)
Cost of the service per child completing the intervention	£1,312 (SD £1342, n= 15)

Table 2: Reach of the Service

	Mean (SD, n)
Potential reach of the service (presuming maximum capacity was achieved) to overweight or obese children within a LA	3.5% (SD 6.9%, n=26) *
Estimated actual reach of the service (i.e. children completing the intervention) to overweight or obese children within a LA	1.2% (SD 1.6%, n=25) *

** These calculations used estimates of the prevalence of overweight or obese children within a LA aged 4-16 years (this was estimated using NCMP data from Reception and Year 6 and National Statistics population data for children aged 4-16 years).*

Qualitative Data from Telephone Interviews

Twenty telephone interviews were conducted with LAs (18 commissioners, 2 service providers within the LA – Interview number 18 and 20). Seventeen of the telephone interview participants had completed the online survey. Three had declined. The geographical location of the twenty LAs who completed the interview were; North West (n=8), North East (n=1), Yorkshire and the Humber (n=1), West Midlands (n=0), East Midlands (n=1), East of England (n=1), London (n=4), South West (n=3) and South East (n=1). Interviews were between 28 and 68 minutes in length.

Nature of Commissioning Contracts

Tier 2 weight management contracts were either between the LA and an external provider, or 'in-house' contracts (where the LA acts as both the commissioner and service provider). Some LAs reported running 'in-house' contracts as they could not afford to commission the service to an external provider. This was not a problem if the service was performing well, however if the service was underperforming, their options might be limited as they may not be able to go out to market due to financial and political pressures.

- **INT 3**: *'if they're not achieving their targets, they're not doing their job properly, so then we shouldn't be providing the service, but what is the alternative? It's too expensive to commission it out'*

One LA discussed the challenges of 'in-house' contracts from a leadership perspective, especially as their service was not meeting BMI targets.

For peer review only - http://bmjopen.bmj.com/site/about/guidelines.xhtml

- **INT 17:** *'To make it complicated our provider is also within the LA so there's a bit of – it's something that provides such a huge challenge just on its own because you've got provider senior leadership and commissioning senior leadership with different views'……… 'the service underachieved against the targets around BMI consistently over the last two years……..If they were an external provider it would probably be a different scenario'*

Outcome Data

All LAs collected outcome data through performance management processes and some also collected outcome data through service evaluation. Most interventions were around 12 weeks long with data collected at baseline and at the end of the intervention. Some LAs also attempted to collect longer-term data at 3 month, 6 months and/or 12 months. Although the general themes of data collected were similar (demographic data, retention, engagement, weight, self-esteem, confidence, behavioural change, physical activity, diet), the actual data were collected in different formats across some LAs. For example, some LAs measured physical activity via a seven-day recall questionnaire, others through a physical activity test and others by asking parents whether their children increased their activity levels or through assessing physical literacy.

Challenges identified through Service Evaluation and/or Performance Management Meetings

1. Lack of long-term data

Many participants mentioned the difficulties in collecting long-term follow-up data. This was attributed to a variety of factors including length of questionnaires, lack of parental confidence with the paperwork, too much effort for families to undertake, people moving around town, resource constraints of LA to capturing this data, lack of IT infrastructure and lack of engagement in both the intervention and the evaluation.

- **INT 14:** *'It becomes then quite time consuming to try and chase patients who engaged. People forget what they've done 12 months ago or more as well. …………it would be quite difficult with not having things like a GP surgeries infrastructure like EMIS where data gets held for years and years and it's there to use and accessed again'.*

2. Lack of validated tools

Some participants felt that there was a lack of validated tools to enable accurate outcome measures to be obtained.

- **INT 1:** *'We're looking for validated tools but there are just not that many great ones out there.'*

3. Reliability of self-report data

For peer review only - http://bmjopen.bmj.com/site/about/guidelines.xhtml

A few participants questioned the reliability of self-report data.

- **INT 3:** *'Other challenges are self-reporting. …. The physical activity and nutrition tend to be improved after ten weeks and sometimes you look on that a little cynically because the measurements haven't improved, so perhaps they're telling us what we want to hear, that can be a challenge'.*

4. Lack of engagement

Difficulties engaging children, parents and healthcare professionals with the service was mentioned by many of the LAs. This is summarised in Table 3.

Table 3: Challenges of Engaging Parents, Children and Health-Care Professionals with the service

Difficulties engaging parents	
Talking about the weight of a child can be highly emotive for parents;	**INT 19:** *'It's difficult with parents sometimes to explain to them that what they are doing at home is probably not the best thing for their child. That's quite difficult you know, that's their baby that's their child and they don't want to hear anything negative.'*
Parents often find it difficult to accept that their child is overweight;	**INT 5:** *'Parents often see their children as normal weight when they are in fact overweight and we know people often will refer to children who are a normal weight as a bit skinny,'*
Parents often do not recognise the role they need to play in engaging in the service as part of a 'family intervention' to improve their child's BMI centile	**INT 11:** *'So we say it has to be a family intervention. But they don't always see it that way. They just want the child to lose the weight and don't acknowledge their role in being the providers' food and the environment they grow up in'.*
Difficulties engaging children	
Engaging children with the service could be challenging;	**INT 2:** *'there is a lot of issues around recruitment and retentions with tier two services for children and also there's a great difficulty with actually the secondary aged children to get them sort of accessing services'.*
Difficulties engaging healthcare professionals	
Healthcare professionals can find it difficult to bring up weight status of a child with a parent.	**INT 4:** *'I think there's definitely issues there from what I've heard about professionals bringing things up with families'*

For peer review only - http://bmjopen.bmj.com/site/about/guidelines.xhtml

Some healthcare professionals fail to recognise overweight or obese children	**INT 5:** *'The GP will look at the child and say, it's just puppy fat, they'll grow out of it'.* **INT 11:** *'We even get some out of school nurses say 'well, they're only just into the overweight category'. You know, the child is really athletic, they're really muscular'*
Lack of GP engagement	**INT 10:** *'GP's still struggle to engage with it'* **INT 6:** *'GPs, locally they tend not to refer'*

5. Lack of resources / expertise

A few commissioners felt that service providers lacked expertise in conducting service evaluations.

- **INT 14:** *'there's difficulties there with the data that we need because we also find that the skill set of a lot of the people delivering the services doesn't always sit with evaluation'.*

Financial Pressures on Services

There are considerable financial pressures facing LAs at present and budget constraints are impacting on the provision of tier 2 weight management services for children in most LAs in different ways.

- **INT 17:** *'we're at a point now where we're going through council budget savings, the service has actually taken a 50% hit, which is huge'.................' so how are we supposed to achieve this whole you know like city wide target on less money is going to be impossible'.........'We've got smaller and smaller services and you keep telling me you're going to take some more money away from me so how are we supposed to achieve these things'*

Some LAs have found it challenging to provide a good service with reduced funding. Strategies taken to cope with the funding cuts have included setting lower targets as part of the key performance indicators (KPIs).

- **INT 1:** *'we've had to work together to reduce the KPIs anyway because they just wouldn't be met with that much of a dent in the finances'*

A few LAs are considering, or have already decided, to decommission their weight management service.

- **INT 2:** *'. So yeah things are really tight and at the regional network meeting people were talking that they may have to de-commission their weight management services'.*

For peer review only - http://bmjopen.bmj.com/site/about/guidelines.xhtml

LAs talked about the need to demonstrate 'good value for money' for a service to justify its funding.

- **INT 5:** *'I'm constantly looking at a cost benefit analysis and working out, okay how much is this costing per child, how much is it costing per family? What are the outcomes that we're getting? Is this really a programme that is cost effective?'*

A few LAs discussed the difficulties in allocating money to service evaluation when money for service provision itself was so limited.

Pressures on Service to Influence the Prevalence of Obesity

LAs often described the pressures they are under to reduce the prevalence of obesity within their borough through their tier 2 weight management programme. In some LAs, this seemed to be politically driven by councillors.

- **INT 2:** *'They're fixated about our actual prevalence rate'…..'the councillors yeah and sort of senior management. We've got like sort of corporate score card and they wanted to put obesity prevalence as part of that.'*

Reducing the prevalence of obesity was frequently seen as an unrealistic goal given the reach of the service often being so small, the funding allocated limited and the feeling that one service cannot be accountable for solely tackling such a complex problem with a programme length that is usually only 10-12 weeks.

- **INT 14;** *'In terms of tackling childhood obesity I'd say the child weight management programmes are family weight management programmes, they're only going to go so far. We know our population in LA14, we've probably got 500 families within each year group that would be affected by obesity even more that would be affected by overweight. If you times that by 18 years of childhood you've got quite a significant number of families up in the 10,000 maybe that are going to have these weight management issues. We're never going to be able to commission a service that would be able to work at a one to one level or a group level with 10,000 families, it's not going to be practical to do that. On the other side of things, we're looking at strategies that take a much more preventative approach.'*

To achieve the objective of reducing the prevalence of obesity, some LAs recognised that population-based approaches would be required.

- **INT 15:** *'the number of people we're getting to is actually quite small …….. it's not going to change obesity levels locally, so we do need to look at more population-based approaches so that's something we will be doing... I suppose doing less programmes possibly in future becaue the numbers per programme aren't as high as we'd want'*

For peer review only - http://bmjopen.bmj.com/site/about/guidelines.xhtml

Need for a 'Whole Systems Approach'

Many of the LAs talked about a recent shift towards a 'whole systems approach' to tackling obesity [21 22 23] and the need to view weight management schemes alongside the 'bigger picture'.

- **INT 2:** *'we can run weight management schemes and I think they're really important, but it has to be part of the bigger picture because you know children's families only go to those sort of schemes like once a week. It's their whole environment that it's important to actually help them to making behaviour change and actually if we don't do both and try and change the obesogenic environment people aren't going to be successful in weight management and it's only going to be a short term, isn't it'*

Some felt that national strategies to try and change the obesogenic environment (e.g. active transport, sugar tax, change for life campaign) and perception of what constitutes a healthy weight were needed to influence the prevalence of obesity.

- **INT 3:** *'It's not going to be easy because it's more and more difficult to make healthy living the norm because it's just too easy to be unhealthy. It's going to take a major upheaval for it to get any better. I think the sugar taxes could help, I think we're going to see more and more of these. What I think we could do to improve it is more and more national campaigns, that's what I think'.*

Sharing and Use of Evaluation Data

Most LAs showed willingness to share data, however this tended to happen on an informal 'when requested' basis. Some LAs reported sharing data with other LAs more formally through obesity network meetings or emails, but this was on a regional rather than national level. Suggestions for future sharing mainly focused on developing online networks, forums or webinars which would enable data to be accessed both on a regional and national level.

- **INT 13:** *'I know in the sexual health areas they have like a forum or something, a website and they all sort of meet up and share best practice and they can ask questions online and things like that, so something like that for weight management would be good'*
- **INT 14:** *'I think there could be like a national monitoring …………………It would be useful to be able to know exactly what data is needed and have methods for having that all collected in one place by one system and then to be able to pull reports from that system locally, regionally, sub-regionally, nationally and even if we could go down to a very local level even a ward level'.*

Some LAs felt that regional and national child obesity commissioner meetings would be useful. A few barriers mentioned to sharing data included time pressures, the commercially sensitive nature of some information and potential competition between LAs, though most did not feel that the latter was a significant issue.

For peer review only - http://bmjopen.bmj.com/site/about/guidelines.xhtml

Within LAs, evaluation or performance management data was mainly used to reshape and improve services and sometimes to promote the service and secure future funding.

Future Directions

1. Guidance needed on service specifications and contracts

Many LAs commented on the lack of consistency in service provider contracts, specifications and outcomes measured across different LAs. They felt that detailed practical guidance with sample service specifications and service provider contracts would be useful, including detailed guidance on what exactly the service should be aiming for in terms of weight loss and other objectives.

- **INT 19**: *I mean there's no like commissioning guidance on weight management programmes you know if that appears on my desk I'd be a very happy bunny because you know then it will tell me exactly what I need to look for, exactly what needs to be achieved. But we don't have a guidance that tells us that you know this is what you should expect from your provider.*
- **INT 17**: *I know trying to find some sort of consistency I think from a contracts point of view, it's been helpful that in other services, not children's weight management where we have had collaborative working around specifications and contracts and then obviously their local detail has been added to it.*

2. Cost Benefit Analyses Tool

In the current economic climate, a few LAs suggested that it would be helpful if researchers developed a cost-benefit analysis tool which they could use for their child weight management programmes to justify allocation of money to these programmes.

- **INT 18:** *'a cost analysis tool. So, in terms of if X loses 5% in terms of weight loss, what that saves NHS/CCG/whoever it may be long term, because we have these cost analysis tools for *another service within the LA*, we have GP cost per hour, things like that, but we don't have anything for weight management for young people, but a cost analysis tool would be great'.*

For peer review only - http://bmjopen.bmj.com/site/about/guidelines.xhtml

Discussion

Main Findings

Data from the online survey demonstrated that no LAs were aware of any peer-reviewed evidence supporting the effectiveness of their specific tier 2 weight management service at improving BMI centile. Service evaluations were not consistently conducted. There was little consistency in methods for reporting change in weight status. The mean cost of the service per child completing the intervention was £1,312 and the mean actual reach of the service (i.e. children completing the intervention) to overweight or obese children within a LA was only 1.2%.

The qualitative research revealed the complexities of 'in-house' contracts in some LAs. There were similarities between LAs in the length of the intervention programme commissioned, the timing of data collection points and the outcomes measured. There were inconsistencies in the tools used to measure these outcomes which complicates meaningful comparisons of data between LAs. Formal sharing of data between LAs was lacking. LAs identified many challenges facing their service in both provision, through lack of engagement and lack of resources, and in-service evaluation, through the questionable reliability of self-report data, lack of validated tools and difficulties in collecting long-term data.

Many LAs described the pressure on their service to reduce the prevalence of obesity but felt that a 'whole systems approach' was needed to tackle this problem rather than over-reliance on a single service. Some LAs felt more detailed guidance was needed on service specifications and contracts. Development of a cost-benefit analysis tool was also discussed by a few LAs.

Meaning of the Findings: Implications for policy makers and clinicians

There is currently no way of easily comparing BMI z-score or other outcome data between different tier 2 weight management programmes across multiple LAs in England. Although PHE have recently developed data entry forms, there is no mandatory system in place requiring LAs to submit this information so it can be collated onto a central database for analysis [24]. Where data are shared, this is usually done on an informal basis at a local level. This is surprising given that the online survey highlighted that no LAs knew of any peer-reviewed evidence supporting the effectiveness of their service at influencing weight status. In addition, there are very few UK-based research trials demonstrating a clinically significant reduction in BMI z-score in school aged children (defined as mean BMI SDS reduction of $\geq$ 0.25) [25-27]. A recent systematic review by Burchett et al reported only five of the thirty interventions included in the review reduced BMI z-score by $\geq$ 0.25 [26]. Of these five interventions, none was conducted in the UK and only 1 involved children of school-age.

Given the current economic climate and lack of evidence regarding long-term effectiveness of these interventions, it would seem wise to ensure that outcome data were being collected in a standardised format and that these data were compared and shared. This could help local and national agencies such as PHE to make evidence-based, cost-effective

For peer review only - http://bmjopen.bmj.com/site/about/guidelines.xhtml

commissioning decisions as the data in this paper suggests that these decisions are currently being conducted without good quality evidence of long-term benefit. However, even if this was achieved, many LAs have already alluded to the difficulties in collecting long-term data and so it is likely that there would be important gaps. It is also plausible that where long term data are collected, no long-term effectiveness is demonstrated. This is possible given that the NICE evidence review supporting the PH47 guideline reported no statistically significant mean difference in BMI z-score in the long-term for lifestyle weight management interventions for children [17].

Many LAs discussed the pressures on their service to reduce the prevalence of obesity. However, the actual mean reach of a service (i.e. children completing the intervention) to overweight or obese children within a LA was 1.2%. It is therefore unrealistic to expect these services to influence obesity prevalence rates. Population measures are needed to have population level effects and it is therefore unclear where Tier 2 services such as those evaluated fall within the overall obesity strategy as they are neither population focussed nor have a strong evidence base for clinically defined groups. Even if the service had the capacity to take a large proportion of the overweight or obese population, the programme still probably would not reach most of this population due to the difficulties in engagement discussed by LAs in the telephone interviews. Problems engaging families with services have been recognised in the literature [28]. Many LAs described the need for a 'whole-systems approach' to effectively tackle the problem of childhood obesity.

A whole systems approach recognises the need to address a complex multi-causal problem using multiple different approaches rather than through a single intervention alone [29 30 31]. On a LA level, this may involve influencing and linking multiple sectors such as planning, housing and transport, to effect population level changes [30 31]. Allender et al describe a community's understanding of the complex causality of obesity through a causal loop diagram [32] and they outline an obesity prevention trial aiming to use a whole systems community-led approach [33]. PHE have commissioned Leeds Beckett University to identify ways in which LAs might achieve a successful whole systems approach [31].

Weaknesses

The sample size for the online survey and telephone interviews were relatively small, but there was good geographical representation across England and saturation was felt to have been achieved in the telephone interviews. It is also not mandatory for LAs to commission a tier 2 weight management service, so some LAs may have felt this research was irrelevant. Due to the method of recruitment to our study, it is possible that in some LAs, details regarding the online survey did not reach the relevant person. A freedom of information (FOI) request was not submitted to obtain missing data and this is a limitation of the study. No implementation theories were used to evaluate programmes.

Although a topic guide was used for the interviews, further discussions were guided by the participant. This had the strength of allowing inductive analyses to be conducted but the weakness that the opinions of every interview participant on each of the themes reported may not have been captured. It is also important to note that the current study focused on

For peer review only - http://bmjopen.bmj.com/site/about/guidelines.xhtml

LAs in England. This means that the generalisation of results to the rest of the UK and wider is unclear.

Finally, LAs did not provide answers in a comparable format for all questions on the online survey which limited statistical analyses to a relatively small number of LAs. This was likely in part due to variation in the type and format of data collected by each LA. A recent PHE study also recognised this problem, reporting that the average change in BMI centile post programme and at 12 months could not be determined due to the heterogeneity of respondents [34]. To gain a true oversight of the cost-effectiveness of lifestyle weight management programmes currently commissioned in the UK, there needs to be consistency in the outcomes measured and clear guidelines on what clinically significant outcomes these services should be aiming to achieve.

Strengths and Contextualisation

In 2015, PHE conducted a national mapping study of tier 2 and tier 3 weight management services [34]. The evidence-base for the commissioned service was determined by asking LAs if they followed NICE guidance or not, rather than asking whether their commissioned service had evidence supporting effectiveness in the peer-reviewed literature, as in this study. This is an important distinction as using guidelines to facilitate commissioning decisions is different to demonstrating the effectiveness of a commissioned service, especially given the limitations of the evidence supporting the NICE PH47 guidelines [35 36].

The PHE mapping study stated that the most frequently reported cost per participant of the service was ≥£401 though there is no further breakdown on figures above this range nor any standard deviations or mean costs provided. As a result, it is not possible to estimate the cost-effectiveness of these interventions. Furthermore, the survey asks for the 'average cost of the intervention per participant' but does not specify whether this should be per participant referred, per participant starting the intervention or per participant completing the intervention [37]. The strengths of our study are that we distinguish costs between these groups and report their means (with standard deviations).

In order to determine whether participants are followed up long-term, the PHE mapping study asked 'How long are the providers required to follow up the participants?' The study reported that 67% of services reported follow-up of participants for 12 months or more. However, being 'required' to follow-up doesn't mean that the data were collected for all these participants. Our qualitative data provides insight into the difficulties in collecting long-term data even when the specification to do so is present.

The qualitative aspect of the PHE study had some similarities with our research, reporting lack of evidence of long-term effectiveness, lack of validated tools, lack of clear guidance on specifications, lack of funding, lack of expertise and difficulties with recruitment.

Future Directions

In their present format, tier 2 weight management services for overweight and obese children are very unlikely to have any impact on the prevalence of childhood obesity and peer-reviewed evidence of any long-term benefits even for the small numbers of children

For peer review only - http://bmjopen.bmj.com/site/about/guidelines.xhtml

reached by these services, is weak. If these lifestyle weight management services are to be continued, clear thought needs to be given to the goals of the service and a more robust independent system needs to be developed to determine whether these goals are being met, whether the service is cost-effective and if it is the best use of limited resources in the current economic climate. Subsequently, if cost effectiveness is demonstrated, work needs to be undertaken to understand the variation in the provision of these services across England, such as through an 'Atlas of Variation [38],' and how LAs can be supported in the commissioning and delivery of these services, given that they are non-mandatory.

However, it is also important to consider whether preferential investment should be given to population level approaches or to developing strategies to deliver a whole systems approach by LAs rather than investing in a single small-scale, lifestyle weight management programme. Population measures such as the sugar tax, have been identified as having the potential to reduce the prevalence of obesity with the greatest benefit predicted for those under the age of 18 [39]. In Mexico, the tax on sugar sweetened beverages (SSBs) in 2013 was associated with fewer taxed beverages being bought and more untaxed beverages being bought [40]. A similar tax in California reduced SSB consumption in low-income neighbourhoods [41]. Yet, there is limited direct evidence of a link between a sugar tax and reduction in obesity prevalence aside from modelling studies. Other population level strategies include reduction of TV advertising of high fat and/or high sugar foods and drinks to children [42], nutritional labelling of foods, transport policies and multi-component mass media campaigns [43]. Nonetheless, Mckinsey et al suggest that public health campaigns have the least evidence for cost effectiveness [44].

Regardless of how funding is allocated to tackling obesity, there needs to be robust cost-effectiveness analyses and sharing of data nationally to help inform future commissioning decisions and ensure that scarce financial resources are being used in the most efficient and effective way across England.

Conclusion

Our results show that none of the participating LAs were aware of any peer-reviewed evidence supporting the effectiveness of their specific commissioned service. Despite this, there was no national formal sharing of data to enable oversight of the effectiveness of commissioned services across LAs in England to help inform future commissioning decisions. Challenges with long-term data collection, service engagement, funding and the pressure to reduce the prevalence of obesity were frequently mentioned. The need for a 'whole-systems approach' to tackle obesity effectively was discussed. In the future, obesity treatment or prevention programmes need to have robust systems in place to feedback programme outcomes and costs in a comparable and transparent format to enable national, independent oversight of the cost-effectiveness of different obesity strategies and direct future commissioning decisions.

Contributors and Guarantor: JHS, RJ, DS, RK and RM conceived and designed the study. Provisional analyses of the online survey data were conducted by RM and the telephone interview data by RM and AP. The results and analyses were reviewed and discussed by JHS,

For peer review only - http://bmjopen.bmj.com/site/about/guidelines.xhtml

RJ and DS. RM drafted the manuscript. All authors revised and approved the final manuscript. The corresponding author attests that all listed authors meet authorship criteria and that no others meeting the criteria have been omitted. RM is guarantor.

Acknowledgements: RM is funded by the NIHR (NIHR ACF 2014-2017, NIHR In-Practice Fellowship 2018-2021). RK works in the Centre for the Development and Evaluation of Complex Interventions for Public Health Improvement (DECIPHer), a UKCRC Public Health Research Centre of Excellence: joint funding (MR/KO232331/1) from the British Heart Foundation, Cancer Research UK, Economic and Social Research Council, Medical Research Council, the Welsh Government and the Wellcome Trust, under the auspices of the UK Clinical Research Collaboration. J.H-S. is supported by the National Institute of Health Research (NIHR) Biomedical Research Centre at the University Hospitals Bristol National Health Service (NHS) Foundation Trust and the University of Bristol, Bristol, UK. DS works in in CAPC PHS funded by HEE Severn.

Funding: Dr R Mears is funded by a National Institute for Health Research (NIHR) Academic Clinical Fellow (ACF-2012-25-009) and In-Practice Fellowship Award (NIHR-IPF-16-10-07) for this research project.

Disclaimer: This publication presents independent research funded by the National Institute for Health Research (NIHR). The views expressed are those of the authors and not necessarily those of the NHS, the NIHR or the Department of Health and Social Care.

Copyright and Licence: The Corresponding Author has the right to grant on behalf of all authors and does grant on behalf of all authors, a worldwide licence (http://www.bmj.com/sites/default/files/BMJ%20Author%20Licence%20March%202013.doc) to the Publishers and its licensees in perpetuity, in all forms, formats and media (whether known now or created in the future), to i) publish, reproduce, distribute, display and store the Contribution, ii) translate the Contribution into other languages, create adaptations, reprints, include within collections and create summaries, extracts and/or, abstracts of the Contribution and convert or allow conversion into any format including without limitation audio, iii) create any other derivative work(s) based in whole or part on the on the Contribution, iv) to exploit all subsidiary rights to exploit all subsidiary rights that currently exist or as may exist in the future in the Contribution, v) the inclusion of electronic links from the Contribution to third party material where-ever it may be located; and, vi) licence any third party to do any or all of the above. All research articles will be made available on an open access basis (with authors being asked to pay an open access fee—see http://www.bmj.com/about-bmj/resources-authors/forms-policies-and-checklists/copyright-open-access-and-permission-reuse). The terms of such open access shall be governed by a Creative Commons licence—details as to which Creative Commons licence will apply to the research article are set out in our worldwide licence referred to above.

Competing Interest Statement*:* All authors have completed the ICMJE uniform disclosure form at www.icmje.org/coi_disclosure.pdf. During the conduct of this study, Dr. Mears and Professor Hamilton-Shield reports grants from National Institute of Healthcare Research,

For peer review only - http://bmjopen.bmj.com/site/about/guidelines.xhtml

Professor Jago reports grants from NIHR and Dr. Kipping reports grants from DECIPHer, grant funding from a collection of charities and funding councils as outlined in the acknowledgments section of this paper. Professor Sharp and Ms Patel have nothing to disclose.

Data Sharing Statement: All data relevant to the study are included in the article or uploaded as supplementary information. Participants gave consent for collection of data which would then be anonymised, so it is not be possible to share individual LA data.

For peer review only

References

1. Collaboration NCDRF. Worldwide trends in body-mass index, underweight, overweight, and obesity from 1975 to 2016: a pooled analysis of 2416 population-based measurement studies in 128.9 million children, adolescents, and adults. *Lancet* 2017;390(10113):2627-42.
2. World Health Organisation. Tenfold increase in childhood and adolescent obesity in four decades: new study by Imperial College London and WHO http://www.who.int/mediacentre/news/releases/2017/increase-childhood-obesity/en/2017 (Accessed March 2019).
3. World Health Organisation. Global Health Observatory Data http://www.who.int/gho/ncd/risk_factors/overweight_obesity/overweight_adolescents/en/2016 (Accessed March 2019).
4. Singh AS, Mulder C, Twisk JW, et al. Tracking of childhood overweight into adulthood: a systematic review of the literature. *Obesity reviews : an official journal of the International Association for the Study of Obesity* 2008;9(5):474-88. doi: 10.1111/j.1467-789X.2008.00475.x [published Online First: 2008/03/12]
5. Finkelstein EA, Graham WC, Malhotra R. Lifetime direct medical costs of childhood obesity. *Pediatrics* 2014;133(5):854-62.
6. NICE. Weight management: lifestyle services for overweight or obese children and young people. PH47. https://www.nice.org.uk/guidance/ph47/history2013 (Accessed March 2019).
7. NHS England. Report of the working group into: Joined up clinical pathways for obesity https://www.england.nhs.uk/wp-content/uploads/2014/03/owg-join-clinc-path.pdf2014 (Accessed March 2019).
8. NHS. NHS structure explained https://www.nhs.uk/using-the-nhs/about-the-nhs/nhs-structure-explained/2016 (Accessed March 2019).
9. HM Government. Health and Social Care Act 2012: structures factsheet https://assets.publishing.service.gov.uk/government/uploads/system/uploads/attachment_data/file/138258/A3.-Factsheet-Overview-of-health-and-care-structures-240412.pdf2012 (Accessed March 2019).
10. NHS Digital. National Childhood Measurement Programme England 2014-2015 https://digital.nhs.uk/catalogue/PUB19109 (Accessed March 2019).
11. Public Health England. Weight management: guidance for commissioners and providers https://www.gov.uk/government/collections/weight-management-guidance-for-commissioners-and-providers2017 (Accessed March 2019).
12. Ford AL, Hunt LP, Cooper A, et al. What reduction in BMI SDS is required in obese adolescents to improve body composition and cardiometabolic health? *Archives of Disease in Childhood*;95(4):256-61.
13. Reinehr T, Lass N, Toschke C, et al. Which Amount of BMI-SDS Reduction Is Necessary to Improve Cardiovascular Risk Factors in Overweight Children? *Journal of Clinical Endocrinology & Metabolism*;101(8):3171-9.
14. Peirson L, Fitzpatrick-Lewis D, Morrison K, et al. Treatment of overweight and obesity in children and youth: a systematic review and meta-analysis. *CMAJ open*;3(1):E35-46.
15. Seburg EM, Olson-Bullis BA, Bredeson DM, et al. A Review of Primary Care-Based Childhood Obesity Prevention and Treatment Interventions. *Current Obesity Reports*;4(2):157-73.
16. Upton P, Taylor C, Erol R, et al. Family-based childhood obesity interventions in the UK: a systematic review of published studies. *Community Practitioner*;87(5):25-9.
17. F. Morgan AW, S. Whitehead, S. Brophy, H. Morgan, R. Turley, R. Kipping, J. White, W. Hollingworth Managing overweight and obesity among children and young people: lifestyle weight management services. Review 1: Effectiveness and cost effectiveness of lifestyle weight management services for children and young people. *NICE* 2013
18. The Kings Fund. Spending on public health https://www.kingsfund.org.uk/projects/nhs-in-a-nutshell/spending-public-health2018 (Accessed March 2019).

For peer review only - http://bmjopen.bmj.com/site/about/guidelines.xhtml

19. National Child Measurement Programme. NCMP- England, 2014-15: Tables https://digital.nhs.uk/data-and-information/publications/statistical/national-child-measurement-programme/2014-15-school-year2015 (Accessed March 2019).
20. Morse JM. "Data were saturated . . . ". *Qualitative Health Research* 2015;25(5):587-8.
21. Local Government Association. Whole Systems Approach to Obesity: Co-creating a world class solution to a world wide challenge https://www.local.gov.uk/sites/default/files/documents/P11%20Using%20whole%20systems%20approaches%20to%20tackle%20obesity.pdf (Accessed March 2019).
22. Local Government Association. Making obesity everybody's business: A whole systems approach to obesity https://www.local.gov.uk/making-obesity-everybodys-business-whole-systems-approach-obesity2017 (Accessed March 2019).
23. House of Commons Health Committee. Eight Report of Session 2017-19. Childhood Obesity: Time for Action https://publications.parliament.uk/pa/cm201719/cmselect/cmhealth/882/882.pdf2018 (Accessed March 2019)
24. Public Health England. Child weight management services: collect and record data. Capturing data: a tool to collect and record child weight management services data. https://www.gov.uk/government/publications/child-weight-management-services-collect-and-record-data2017 (Accessed March 2019).
25. Al-Khudairy L, Loveman E, Colquitt JL, et al. Diet, physical activity and behavioural interventions for the treatment of overweight or obese adolescents aged 12 to 17 years. *Cochrane Database of Systematic Reviews*;6:CD012691.
26. Burchett HED, Sutcliffe K, Melendez-Torres GJ, et al. Lifestyle weight management programmes for children: A systematic review using Qualitative Comparative Analysis to identify critical pathways to effectiveness. *Preventive Medicine*;106:1-12.
27. Mead E, Brown T, Rees K, et al. Diet, physical activity and behavioural interventions for the treatment of overweight or obese children from the age of 6 to 11 years. *Cochrane Database of Systematic Reviews*;6:CD012651.
28. Kelleher E, Davoren MP, Harrington JM, et al. Barriers and facilitators to initial and continued attendance at community-based lifestyle programmes among families of overweight and obese children: a systematic review. *Obesity Reviews*;18(2):183-94.
29. Lee BY, Bartsch SM, Mui Y, et al. A systems approach to obesity. *Nutrition Reviews*;75(suppl 1):94-106.
30. Rutter H, Savona N, Glonti K, et al. The need for a complex systems model of evidence for public health. *Lancet*;390(10112):2602-04.
31. Leeds Beckett University. Whole Systems Approach to Tackle Obesity http://www.leedsbeckett.ac.uk/wholesystemsobesity/: Leeds Beckett University (Accessed March 2019).
32. Allender S, Owen B, Kuhlberg J, et al. A Community Based Systems Diagram of Obesity Causes. *PLoS ONE [Electronic Resource]*;10(7):e0129683.
33. Allender S, Millar L, Hovmand P, et al. Whole of Systems Trial of Prevention Strategies for Childhood Obesity: WHO STOPS Childhood Obesity. *International Journal of Environmental Research & Public Health [Electronic Resource]* 2016;13(11):16.
34. Public Health England. National Mapping of Weight Management Services, 2015 https://assets.publishing.service.gov.uk/government/uploads/system/uploads/attachment_data/file/484115/Final_Weight_Management_Mapping_Report.pdf (Accessed March 2019).
35. Morgan F WA, Whitehead S, Brophy S, Morgan HE, Turley RL, Kipping R, White J, Hollingworth W. . Managing overweight and obesity among children and young people: Review 1 - Effectiveness and cost effectiveness of lifestyle weight management services for children and young people. In: NICE, ed., 2013.

For peer review only - http://bmjopen.bmj.com/site/about/guidelines.xhtml

36. Turley RL WA, Halstead E, Morgan HE, Morgan F, Noyes J, Brophy S, Kipping R and Whitehead S. . Managing overweight and obesity among children and young people: lifestyle weight management services. Review 2: The barriers and facilitators to implementing lifestyle weight management programmes for children and young people. In: NICE, ed., 2012.
37. Public Health England. National mapping of weight management services: e-survey and face to-face approach, 2015 https://assets.publishing.service.gov.uk/government/uploads/system/uploads/attachment_data/file/768890/National_mapping_of_weight_management_services_Annex_2.pdf (Accessed March 2019)
38. Public Health England. Atlas of Variation https://fingertips.phe.org.uk/profile/atlas-of-variation (Accessed March 2019).
39. Briggs ADM, Mytton OT, Kehlbacher A, et al. Health impact assessment of the UK soft drinks industry levy: a comparative risk assessment modelling study. *The lancet Public Health*;2(1):e15-e22.
40. Colchero MA, Popkin BM, Rivera JA, et al. Beverage purchases from stores in Mexico under the excise tax on sugar sweetened beverages: observational study. *BMJ*;352:h6704.
41. Falbe J, Thompson HR, Becker CM, et al. Impact of the Berkeley Excise Tax on Sugar-Sweetened Beverage Consumption. *American Journal of Public Health*;106(10):1865-71.
42. Haby MM, Vos T, Carter R, et al. A new approach to assessing the health benefit from obesity interventions in children and adolescents: the assessing cost-effectiveness in obesity project. *International Journal of Obesity*;30(10):1463-75.
43. World Health Organisation. Population-based approaches to childhood obesity prevention https://www.who.int/dietphysicalactivity/childhood/WHO_new_childhoodobesity_PREVENTION_27nov_HR_PRINT_OK.pdf2012 (Accessed March 2019).
44. Dobbs R SC, Thompson F, Manyika J, Woetzel J, Child P, Mckenna S and Spatharou A. Overcoming obesity: an initial economic assessment. https://www.mckinsey.com/~/media/mckinsey/business%20functions/economic%20studies%20temp/our%20insights/how%20the%20world%20could%20better%20fight%20obesity/mgi_overcoming_obesity_full_report.ashx: McKinsey Global Institute; 2014 (Accessed March 2019).

For peer review only - http://bmjopen.bmj.com/site/about/guidelines.xhtml

Supplementary File 1 – Online Survey Questions:

National Survey of LA Commissioned Weight Management Services for Overweight / Obese Children in 2014/15

	**Informed Consent for Online Survey**
	In order to take part in this online survey, we need to obtain your informed consent. Please read the following five statements carefully.<br>1. I confirm that I have read and understood the participant information.<br>2. I am willing to take part in the survey<br>3. I understand that my name will be kept anonymous however due to the nature of the study, it is not possible to anonymise the local authority name<br>4. I understand that information collected (name of survey participant anonymised but name of local authority not anonymised) will be stored for 10 years in data sets within a secure facility in accordance with the Data Protection Act 1998 and this data may be used in publications or presentations to relevant audiences or shared with other researchers.<br>5. I understand that I can withdraw from the study at any point prior to March 21st 2016 by emailing Dr Ruth Mears on rm14101@bristol.ac.uk
**Question 1**	Please confirm that you have read, understood and agree to the above five statements
**Response 1**	
	**Participant & Local Authority Details**
**Question 2**	What local authority do you work in?<br>*Please note that if you work for multiple local authorities, you will need to fill out a new survey for each local authority that you work for.*
**Response 2**	
	**Tier 2 Weight Management Services for Overweight/Obese Children in your Local Authority**
**Question 3**	Please name a Tier 2 weight management service commissioned by your local authority for overweight / obese children aged 4-16 years during the financial year April 2014-March 2015?
**Response 3**	
**The following questions relate to the Tier 2 weight management service you have named above.**	
**Question 4**	What evidence are you aware of regarding the effectiveness of the service commissioned between April 2014 – March 2015 at reducing BMI centile / BMI % / BMI z-score or BMI? Please choose (highlight) from the below list;
**Response 4**	– *Data published in a peer reviewed journal – independently collected (i.e. data collected by a person who is NOT an employee of the weight management service provider)*

For peer review only

University of BRISTOL

	– *Data published in a peer reviewed journal – internally collected (i.e. data collected by a person who IS an employee of the weight management service provider)*<br>– *Published in an alternative source – independently collected (i.e. data collected by a person who is NOT an employee of the weight management service provider)*<br>– *Published in an alternative source – internally collected (i.e. data collected by a person who IS an employee of the weight management service provider)*<br>– *Unpublished data*<br>– *Other*<br>– *No evidence*
**Question 5**	Please specify where the evidence can be found regarding the effectiveness of the services commissioned during the year April 2014-March 2015 at reducing BMI centile / BMI % / BMI z-score or BMI?<br>*(e.g. publication details / website address etc. If the data is unpublished, please email details to rm14101@bristol.ac.uk)*
**Response 5**	
**Question 6**	Since the service was commissioned (i.e. contract start date), had it been evaluated within your local authority?
**Response 6**	
**Question 7**	As part of the service evaluation, was change in weight status measured (e.g. change in BMI, BMI%, BMI centile or BMI z-score?)
**Response 7**	
**Question 8**	If change in weight status was measured, what were the results?<br>*Please write the time frame in which this change occurred e.g. Reduction of BMI centile by 0.9% (SD) over 1 year (2014/15). If this is available for different age groups, please indicate the results by age group.*
**Response 8**	
**Question 9**	What was the total cost of the service for the local authority between April 2014 to March 2015?<br>*If data cannot be provided please specify the time period and costs in the format you have available e.g. cost per child per course in August 2014.*
**Response 9**	
**Question 10**	What was the maximum number of participants that could have been accommodated by the commissioned service between April 2014 to March 2015?<br>*Where possible, please provide data on maximum commissioned capacity for a one year time frame from 2014 to 2015. If this data cannot be provided, please specify the time period and maximum capacity of the service in the format you have available e.g. maximum capacity of 60 children per course in 2014, total of 10 courses in 2014.*

For peer review only

**Response 10**	
**Question 11**	How many children were referred to the service between April 2014 to March 2015?<br>*If this data cannot be provided please specify the time period and referral data in the format you have available*
**Response 11**	
**Question 12**	How many children completed the intervention between April 2014 to March 2015?<br>*If data cannot be provided please specify the time period and number completing the intervention in the format you have available*
**Response 12**	
	**Thank you for taking the time to complete this survey. Please consider taking part in the second phase of our research.**
**Question 13**	Thank you for taking the time to complete this survey.<br><br>Would you like to receive a summary of the results and analysis by email?<br><br>If you answered yes to the above question, please provide us with your email address
**Response 13**	
**Question 14**	If you answered yes to the above question, please provide us with your email address
**Response 14**	
	**Telephone Interview**

The second phase of our research will involve a telephone interview exploring commissioners' views and experiences in the evaluation of weight management services for overweight and obese children. There is little qualitative evidence available regarding service evaluation data collected by commissioners and this research aims to fill the gap in the literature. We will explore the views of commissioners on the role and value of service evaluation data, the barriers and facilitators to collecting and processing this data and finally how to ensure evaluation data is useful. Performance management of services will also be explored.

If you are interested in participating in the interview, please can you provide your name and contact details (email and/or telephone number) below. We will then email you a participant information sheet providing further details about what the interview involves. After reading the information sheet, if you decide you would like to take part, you will need to fill out the consent form and send it to rm14101@bristol.ac.uk. Dr Ruth Mears will then contact you to arrange a convenient time for you to conduct the telephone interview

……
……
……

For peer review only

For peer review only

Supplementary File 2

Interview Guide: Commissioning and evaluation of lifestyle weight management programmes in England

**Icebreaker**	**Prompts**
What is your role in the local authority?	*How long have you been in this role?*
How are you involved in the commissioning of weight management services for obese and overweight children?	*What is your background? Public Health?*
**Part One: Role and value of evaluations**	
What do you understand by the term 'service evaluation'?	*Why conduct a service evaluation? Role for commissioners? Role for participants? Role for service providers?*
What do you understand by the term 'performance management'?	*Why collect performance management data?*
How do you think service evaluation and performance management differ?	
How important do you think service evaluation is?	*Importance to commissioners / LA / personal opinions? Why? What informs the decision to undertake a service evaluation (e.g. pilot, new service, lack of evidence, review, retenderin)?*
How useful do you think service evaluation is?	*Why? What parts are useful? What parts are not useful? Do your personal views differ from what you feel are the views of the local authority?*
What outcomes are currently viewed as a measure of success by your local authority for a childhood weight management programme?	*Do you agree that this outcome should be viewed as a measure of success? What are your personal views? Which outcomes do you think are the most important? Why?*
What outcome data from a service evaluation is viewed as essential by your local authority? *(or performance management data if service evaluations have not been conducted)*	*Why? Do you agree?*
What information from a service evaluation does your local authority least value? *(or performance management data if service evaluations have not been conducted)*	*Why? Do you agree?*

For peer review only

Supplementary File 2

**Part Two: LA Specific Questions**	
Reflecting on a weight management service you have been involved in, can you tell me how the service was evaluated?<br>*If the service has not been evaluated, please can you tell me about performance management of the service.*	*Who collected the evaluation data (external evaluator or internal person)? At what time points was the data collected? What data was collected? Was a specific evaluation data collection tool used?*
What went well in collecting evaluation data *(or performance management data if no service evaluation has been conducted)*?	*What barriers were there? What are the weaknesses in your data? What are the strengths of your data? Were service providers happy to co-operate with the evaluation process? Were there any difficulties in collecting data from service users? How could service evaluation data be improved?*
What did not go well in collecting evaluation data *(or performance management data if no service evaluation has been conducted)*?	
Do you think the data collected was useful?	***Yes:*** *In what way was it useful? How could it have been more useful? How were the data used?*<br>**No:** *Why do you think it was not useful? Is there are anything which would have made it more useful?*
How were the service evaluation data *(or performance management data if no service evaluation has been conducted)* used by your local authority?	*By commissioners? By service providers? To improve services? To inform future commissioning decisions? Is this reflective of how other service evaluation data has been used*
How do you think the information collected from a service evaluation *(or performance management data if no service evaluation has been conducted)* should be used for maximum benefit?	*By commissioners? By service providers?*
**Part Three: Improving the process of service evaluation and use of outcome data**	
What resources / tools / information / guidance are currently available to help commissioners conduct service evaluations?	*Are they useful? What are the downsides of them? What would you find useful to have which is not currently available?*
Do you have access to online journals - OVID / Medline databases etc?	*Would you feel comfortable reviewing evidence from these databases?*
Who is responsible for collecting the service evaluation *(or performance management)* outcome data in your local authority?	*If it is the service providers, do you think they should be? Why? Why not?*
What are your opinions on the sharing of evaluation *(or performance data)* between local authorities and other organisations?	*Do you have any reservations? Do you think it would be beneficial or*

For peer review only

Supplementary File 2

	detrimental? Use in future commissioning decisions?
Do you share evaluation *(or performance data)* with other local authorities or organisations?	***Yes:*** *How do you do this? Is the data actively shared? Is it useful? Is the data shared of good quality? Does it play a role in future commissioning decisions?*<br>***No:*** *Why not? Do you think data should be shared? What are the barriers to sharing data?*
Do you think service contracts should be based on performance?	*E.g. must attain x change in BMI percentile or the contract will be terminated. What are the benefits of linking service contracts to performance? What are the downsides of this?*
In your Local Authority, are service evaluations used to identify underperforming services?	***Yes:*** *Do you think they should be used in this way? If a weight management service within your local authority is underperforming, are there any procedures which you would follow?*<br>***No:*** *Do you think they should be used in this way?*
How do you think the process of service evaluation *(or performance data)* and use of outcome data could be optimised to ensure maximum benefit for commissioners?	*Maintaining standards? Future decisions? Sharing?*
Are you aware of any recent national (NOO or NICE) guidelines regarding the evaluation of lifestyle weight management programmes?	*What do you know about them? At what time points do these guidelines recommend collecting outcome data? Does your local authority currently collect data at this timepoint? Is your local authority planning to implement these guidelines?*
What information do you think is needed to assist commissioners in providing successful weight management programmes?	*(Prompts; research? evidence? guidelines?)*
**Closing**	
That's all the questions I have for you today. Do you have any other comments you wish to make about service evaluations?	
Do you have any questions for me?	
Thank you very much for your time and attention. We appreciate you sharing your thoughts and opinions with us.	

For peer review only

COREQ (COnsolidated criteria for REporting Qualitative research) Checklist

A checklist of items that should be included in reports of qualitative research. You must report the page number in your manuscript where you consider each of the items listed in this checklist. If you have not included this information, either revise your manuscript accordingly before submitting or note N/A.

Topic	Item No.	Guide Questions/Description	Reported on Page No.
**Domain 1: Research team and reflexivity**			
Personal characteristics			
Interviewer/facilitator	1	Which author/s conducted the interview or focus group?	Pg 3
Credentials	2	What were the researcher's credentials? E.g. PhD, MD	MBBCh, BSc
Occupation	3	What was their occupation at the time of the study?	GP Registrar, NIHR ACF
Gender	4	Was the researcher male or female?	Female
Experience and training	5	What experience or training did the researcher have?	MBBCh, BSc
Relationship with participants			
Relationship established	6	Was a relationship established prior to study commencement?	No
Participant knowledge of the interviewer	7	What did the participants know about the researcher? e.g. personal goals, reasons for doing the research	RM working part-time in general practice and part-time in research
Interviewer characteristics	8	What characteristics were reported about the inter viewer/facilitator? e.g. Bias, assumptions, reasons and interests in the research topic	As above
**Domain 2: Study design**			
Theoretical framework			
Methodological orientation and Theory	9	What methodological orientation was stated to underpin the study? e.g. grounded theory, discourse analysis, ethnography, phenomenology, content analysis	Pg 4, Inductive thematic analysis
Participant selection			
Sampling	10	How were participants selected? e.g. purposive, convenience, consecutive, snowball	Pg 3
Method of approach	11	How were participants approached? e.g. face-to-face, telephone, mail, email	Pg 5
Sample size	12	How many participants were in the study?	Pg 6
Non-participation	13	How many people refused to participate or dropped out? Reasons?	From the online survey, 31 expressed an interest to participate in the telephone interviews.

For peer review only

Setting			
Setting of data collection	14	Where was the data collected? e.g. home, clinic, workplace	Workplace
Presence of nonparticipants	15	Was anyone else present besides the participants and researchers?	Nil with RM. Interview participants may be sharing an office.
Description of sample	16	What are the important characteristics of the sample? e.g. demographic data, date	Pg 6
Data collection			
Interview guide	17	Were questions, prompts, guides provided by the authors? Was it pilot tested?	Pg 3
Repeat interviews	18	Were repeat inter views carried out? If yes, how many?	No
Audio/visual recording	19	Did the research use audio or visual recording to collect the data?	Pg 4
Field notes	20	Were field notes made during and/or after the inter view or focus group?	No
Duration	21	What was the duration of the inter views or focus group?	Pg 6
Data saturation	22	Was data saturation discussed?	Pg 4
Transcripts returned	23	Were transcripts returned to participants for comment and/or correction?	No
**Topic**	**Item No.**	**Guide Questions/Description**	**Reported on Page No.**
**Domain 3: analysis and findings**			
Data analysis			
Number of data coders	24	How many data coders coded the data?	Pg 4
Description of the coding tree	25	Did authors provide a description of the coding tree?	No
Derivation of themes	26	Were themes identified in advance or derived from the data?	Pg 4
Software	27	What software, if applicable, was used to manage the data?	Pg 4
Participant checking	28	Did participants provide feedback on the findings?	Not yet. Summary will be sent to participants in due course.
Reporting			
Quotations presented	29	Were participant quotations presented to illustrate the themes/findings? Was each quotation identified? e.g. participant number	Pg 6-13
Data and findings consistent	30	Was there consistency between the data presented and the findings?	Pg 14-17
Clarity of major themes	31	Were major themes clearly presented in the findings?	Pg 6-13
Clarity of minor themes	32	Is there a description of diverse cases or discussion of minor themes?	Pg 6-13

Developed from: Tong A, Sainsbury P, Craig J. Consolidated criteria for reporting qualitative research (COREQ): a 32-item checklist for interviews and focus groups. *International Journal for Quality in Health Care*. 2007. Volume 19, Number 6: pp. 349 – 357

For peer review only

Once you have completed this checklist, please save a copy and upload it as part of your submission. DO NOT include this checklist as part of the main manuscript document. It must be uploaded as a separate file.

For peer review only
